# Mitochondrial Variability as a Source of Extrinsic Cellular Noise

**DOI:** 10.1371/journal.pcbi.1002416

**Published:** 2012-03-08

**Authors:** Iain G. Johnston, Bernadett Gaal, Ricardo Pires das Neves, Tariq Enver, Francisco J. Iborra, Nick S. Jones

**Affiliations:** 1 Department of Physics, Clarendon Laboratory, Oxford, United Kingdom; 2 Oxford Centre for Integrative Systems Biology, Department of Biochemistry, Oxford, United Kingdom; 3 UCL Cancer Institute, University College London, London, United Kingdom; 4 Center for Neuroscience and Cell Biology, University of Coimbra, Coimbra, Portugal; 5 Biomaterials and Stem Cell-based Therapeutics Group and Biocant – Center of Innovation and Biotechnology, Cantanhede, Portugal; 6 Department of Molecular and Cellular Biology, Centro Nacional de Biotecnología, Consejo Superior de Investigaciones Científicas, Madrid, Spain; 7 Department of Mathematics, Imperial College London, London, United Kingdom; North Carolina State University, United States of America

## Abstract

We present a study investigating the role of mitochondrial variability in generating noise in eukaryotic cells. Noise in cellular physiology plays an important role in many fundamental cellular processes, including transcription, translation, stem cell differentiation and response to medication, but the specific random influences that affect these processes have yet to be clearly elucidated. Here we present a mechanism by which variability in mitochondrial volume and functionality, along with cell cycle dynamics, is linked to variability in transcription rate and hence has a profound effect on downstream cellular processes. Our model mechanism is supported by an appreciable volume of recent experimental evidence, and we present the results of several new experiments with which our model is also consistent. We find that noise due to mitochondrial variability can sometimes dominate over other extrinsic noise sources (such as cell cycle asynchronicity) and can significantly affect large-scale observable properties such as cell cycle length and gene expression levels. We also explore two recent regulatory network-based models for stem cell differentiation, and find that extrinsic noise in transcription rate causes appreciable variability in the behaviour of these model systems. These results suggest that mitochondrial and transcriptional variability may be an important mechanism influencing a large variety of cellular processes and properties.

## Author Summary

Cellular variability has been found to play a major role in diverse and important phenomena, including stem cell differentiation and drug resistance, but the sources of this variability have yet to be satisfactorily explained. We propose a mechanism, supported by a substantial number of recent and new experiments, by which cell-to-cell differences in both the number and functionality of mitochondria – the organelles responsible for energy production in eukaryotes – leads to variability in transcription rate between cells and may hence be a significant source of cellular noise in many downstream processes. We illustrate the downstream effect of mitochondrial variability through simulated studies of protein expression and stem cell differentiation, and suggest possible experimental approaches to further elucidate this mechanism.

## Introduction

Stochastic influences significantly affect a multitude of processes in cellular biology [1]–[5]. Understanding the sources of this randomness within and between cells is a central current challenge in quantitative biology. Noise has been found to affect processes including stem cell fate decisions [6], bet-hedging in bacterial phenotypes [7], [8], cancer development [9], and responses to apoptosis-inducing factors [10], [11]. In this paper, we consider how mitochondria may constitute a significant source of this cellular noise.

Noise in cellular processes may result from sources intrinsic to the gene in question (those responsible for differences in the expression levels of genes under identical regulation in the same cell) or extrinsic sources (those responsible for cell-to-cell variation in genes under identical regulation in a population). Both intrinsic and extrinsic noise sources contribute to the overall noise observed in, for example, transcription rates and protein expression levels [12]. The interplay between intrinsic and extrinsic noise can be characterised with elegant experimental techniques such as dual reporter measurements [3], in which the expression levels of two proteins are measured within cells and within a population, but subtleties exist in disambiguating intrinsic and extrinsic contributions to noise levels [13]. Some studies have found the contribution of extrinsic factors to overall noise levels to be stronger in eukaryotes [14], [15] than prokaryotes [3], although others debate this interpretation [16]. To investigate these influences, several mathematical models for the emergence of intrinsic and extrinsic cellular noise have been introduced and explored [12], [17]–[24]. In addition, recent studies have investigated, both experimentally and theoretically, the architecture of extrinsic noise and its causal factors [14]–[16], [19], [25]–[27], though substantial uncertainty surrounds the importance of individual contributions (such as variability in cell cycle stage and cellular volume) to extrinsic noise [28].

Huh and Paulsson recently argued that uneven segregration of cellular constituents at mitosis can contribute significantly to cell-to-cell differences in levels of cellular components and proteins in a population, focusing on stochasticity in protein inheritance between sister cells [29], [30]. We focus on a specific instance of this phenomenon: cell-to-cell variability in the mitochondrial content of cells. An experimental study performed by das Neves *et al.* identified uneven partitioning of mitochondria at mitosis as being a possibly significant source of extrinsic noise in eukaryotes [31], supporting recent theoretical ideas [30]. Mitochondria have been found to display remarkably complex behaviour interwoven with cellular processes [32]–[34] and to display significant heterogeneity within cells [31], [35]–[37]. Mitochondrial influences on processes including stem cell differentation [38] and cell cycle progression [39]–[41] have recently been observed.

das Neves *et al.* [31] observe a wide spread of mitochondrial masses in a population of cells, illustrating extrinsic variability in organelle distribution. Mitochondrial functionality has also been observed to vary between cells [34], [35], [42]–[44]. das Neves *et al.* also observed a link between mitochondrial mass and membrane potential and cellular ATP levels, and found transcription rate to be a function of ATP concentration. In addition, the modulation of mitochondrial functionality, through anti- and pro-oxidant treatments, was found to alter cell-to-cell variability in transcription rates, with anti-oxidants significantly reducing variability and pro-oxidants increasing variability. These results suggest that cell-to-cell heterogeneity in mitochondrial mass and functionality may propagate into extrinsic noise in transcription rate, and thenceforth processes further downstream, but the quantitative links behind these processes remain unclear. We introduce a simple approach, consistent with a range of experimental observations, that quantitatively connects all these features and predicts the downstream physiological influence of mitochondrial variability.

Shahrezaei *et al.* [45] have recently shown that extrinsic noise can influence levels of intrinsic noise, as cell-to-cell variability in the rates of processes such as transcription and translation affect the intrinsic dynamics of gene expression. In addition, they provided an extension to standard stochastic simulation techniques to allow this variability in the production rates of chemical species to be accurately simulated – a problem that has been approached using different techniques in previous studies [46], [47]. However, this theoretical study did not attempt to characterise the physiological causes of this extrinsic noise – an important consideration in assessing the ubiquity and consequences of cellular noise. Our proposal that cell-to-cell mitochondrial variability provides a significant source of extrinsic noise in transcription addresses these causes, and we show that extrinsic noise resulting from mitochondrial variability could significantly influence intrinsic noise in gene expression.

This paper will proceed as follows. We first introduce one of the simplest possible mathematical models for variation in mitochondrial mass and functionality during and between cell cycles, and show that it is consistent with a wide range of experimental data, both from the literature and newly reported here, and allows analytical treatment. Our model includes stochastic segregation of mitochondria at mitosis and functional differences in mitochondria between cells, and contains a simple dynamic description of the time evolution of cellular volume and mitochondrial mass through the cell cycle. To our knowledge it is the first model of its kind which links mitochondrial mass and function to the cell cycle and gene expression. We relate mitochondrial properties to the production of ATP in the cell, which in turn affects transcription rates: hence, variability in mitochondrial properties causes downstream variability in transcription. Next, we incorporate the behaviour produced by our model into a common framework for cellular noise, and show that extrinsic noise due to variation in 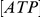 can have a profound effect on gene expression levels, dominating over intrinsic noise. We then demonstrate the cell physiological implications of energy variability by showing how mitochondrial variability may affect stem cell differentation. Finally, we discuss how our model relates to recent work characterising sources of extrinsic noise, and suggest experiments to allow more refined models.

## Results

In this section, we first describe the approach we use to model mitochondrial variability in a population of cells. Next, we compare recent experimental data from Ref. [31] (demonstrating transcription rate variability in a range of cell types and exploring cellular variability in detail in HeLa cells) to the predictions of our model and demonstrate that a good agreement exists across a wide range of experiments. We then report new experimental results of relevance to the study of mitochondrial variability and show that these too largely agree with the predictions from our simple model. This set of successful comparisons suggests that our model is capable of producing quantitatively sound estimates of the levels of noise associated with mitochondrial sources of variability. Motivated by these results, we next show how our model allows a quantitative link to be formed between mitochondrial variability and variability in transcription rate in cells. We explore this link by investigating the predictions that our model makes concerning noise in models of gene expression levels, and in models of stem cell differentiation pathways. We find that the mitochondrial sources of variability from our model could provide a substantial contribution to noise levels in mRNA and protein levels within the cell, and can influence stem cell differentiation in a manner that depends upon the symmetry of the regulatory interactions that drive differentiation.

### Model

While the heterogeneity of mitochondria has been observed experimentally and connected to variability in processes like transcription [31] and stem cell differentiation [38], the mechanisms by which mitochondrial variability influences other cellular processes has not been elucidated clearly. Here, we describe a simple model which formalises these links, and note that it is consistent with recent experimental results concerning mitochondrial heterogeneity (and variability in connected cellular features) [31]. The simplicity of our model means that analytic expressions can be derived for many quantities of interest, facilitating a more complete and intuitive understanding of the modelled biological connections. We will then use this model to investigate more specific questions regarding the links between mitochondrial variability and transcription rate and stem cell differentiation.

**Figure 1:**
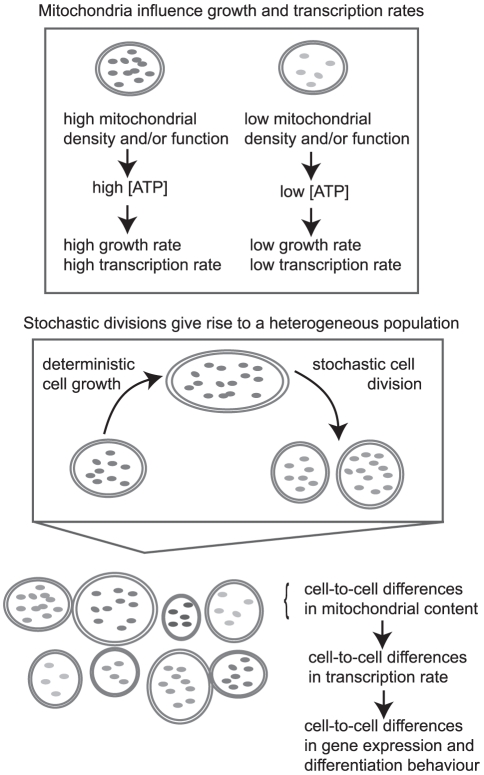
An illustration of the model we employ for mitochondrial variability. This illustration qualitatively shows the key components of our model. Cell growth progresses deterministically according to the variables that characterise a cell: volume, mitochondrial mass (illustrated here by copy number) and functionality (illustrated here by shading). At mitosis, stochastic partitioning occurs and daughter cells inherit a random volume, mitochondrial mass and functionality level from a parent cell. This stochastic inheritance leads to a heterogeneous population. Cells with high mitochondrial density and functionality have higher ATP levels, are able to grow faster, and have higher transcription rates than cells with lower mitochondrial mass and functionality. The variances associated with stochastic partitioning, the dependence of ATP concentration on cellular properties, and the dependence of growth and transcription rates on ATP are all parameters of the model.

The central concept behind our model is illustrated in Fig. 1. Individual cells are characterised by three key variables: the volume of the cell (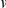); the amount of mitochondrial mass in the cell (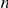); and the degree of mitochondrial functionality (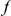). This last quantity, 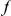, represents a coarse-grained measure of the efficiency of mitochondria within a cell – a factor which may be affected, for example, by the levels of reactive oxygen species (ROS), mitochondrial membrane potential, variability in mitochondrial protein complex abundance, and genetic differences between mitochondria [48]. ATP concentration in the cell is modelled as a function of these three quantities, and transcription rate is modelled as a function of ATP concentration [31]. Variability in cell volume, mitochondrial mass and mitochondrial functionality arises due to stochastic inheritance of these quantities at cell divisions. This variability causes cell-to-cell differences in ATP levels, and hence transcription rate, in a population of cells.

#### Stochastic partitioning at mitosis

In our model, cells grow deterministically (see Cellular Dynamics), undergoing mitosis when their volume reaches a cutoff 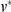. When this occurs, the cell divides in two, with mitochondrial mass 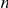 split stochastically between daughter cells, with each unit of mass being assigned to each cell with equal probability, and cell volume also segregated randomly (see Methods). In our model, the partitioning of 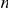 and 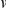 at mitosis is uncorrelated. We use this lack of correlation both for simplicity and due to experimental data (see Results) illustrating that cell cycle length correlates well with inherited mitochondrial mass and poorly with inherited cell volume, indirectly suggesting a lack of correlation between 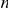 and 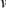. This model was chosen as the most straightforward representation of stochastic division of discrete elements, and is likely to represent a realistic scenario if there is no explicit biological control mechanism that modulates the distribution of inherited mitochondria.

Our mitochondrial mass measure (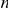) physically represents total mitochondrial volume. However, it will be of use when considering the segregation of mitochondria at mitosis to consider the cell as populated by a number of discrete ‘virtual’ mitochondria. We denote these entities as ‘virtual’ mitochondria due to the difficulty of regarding mitochondria as individuals given the processes of fission and fusion [49]. The system as chosen is scaled so as to regard 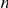 as mitochondrial copy number, so that, if 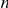 is measured in 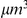, each ‘virtual’ mitochondrion possesses a default volume of 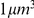 (see Methods). These virtual mitochondria are the discrete elements that, in our model, are binomially partitioned at mitosis – resembling elements of a fragmented mitochondrial network that are split between daughter cells [50]. We use the binomial picture both for simplicity and due to its agreement with recent data on mitochondrial partitioning [31], but note that a range of mitochondrial partitioning regimes have been observed in the literature [29], [51], [52], and explore (in the Results section) the effects of wider or narrower distributions associated with mitochondrial partitioning.

We consider the variable 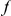 to be the degree of functionality of a cell's mitochondria. The inclusion of such a term is necessitated by several experimental observations. das Neves *et al.* show that a measure of mitochondrial functionality (membrane potential) is slowly-varying with time in a given cell, although there is a wide distribution of functionality within a population [31]. It was also found that sister cells have similar transcriptional noise levels compared to the bulk population: if stochasticity were to arise from mitochondrial mass partitioning alone, we would expect sister cells, post-mitosis, to exhibit the greatest possible variation, as subsequent cell growth may be expected to dampen such variability [30]. Another experimental observation is that populations of cells treated with antioxidants, which improve mitochondrial functionality, showed a significant drop in noise levels. These results suggest that an extra source of noise, functional variability between cells, may be responsible for increasing noise levels.

In the absence of a more refined view of functionality, we assume that all changes in functionality occur at division and that 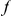 stays constant through the cell cycle. 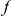 changes in a stochastic but mean-reverting fashion at division, and both daughters receive the same 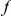 value (see Methods for more detail). In this simple model, the variation that a cell experiences due to slow changes in mitochondrial functionality through the cell cycle is absorbed into stochastic changes at cell division. We choose this modelling protocol due to the absence of detailed data on the behaviour of mitochondrial functionality on timescales longer than a cell cycle, and suggest that parameterising this simple system to match the experimentally observed distribution of mitochondrial functionality will give a reasonable estimate of the population variability in this quantity. In ‘Other Models’ in Text S1, we discuss another picture in which we allow 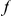 to vary continuously through the cell cycle, and show that similar results emerge when this alternative model is used.

In this study, we will consider the oxidative state of a cell as a key mediator of its functionality 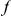. Recent experimental data has shown that treating cells with pro- or anti-oxidants strongly affects the statistics of transcription rate variability in a population [31]. Within our model, the effects of such chemical treatments on the oxidative state of cells can straightforwardly be captured by varying the parameters associated with functional inheritance (see Methods).

#### 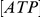 and transcription rate

We are interested in the time evolution of 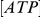 as a potential stochastic influence on downstream processes. Ref. [31] found ATP levels in the cell to be proportional to mitochondrial mass (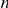) and membrane potential (a factor that may be absorbed into our measure of ‘mitochondrial function’ 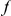), motivating our choice of expression for ATP concentration:
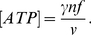
(1)


In this expression, 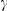 is a constant of proportionality linking the quantities within our model to a biological ATP concentration, and the meaning of the variable 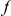 now becomes apparent as a scalar multiple relating mitochondrial density to 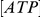. We note that other choices for the form of 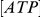, including ODEs, are possible, and explore some alternatives in ‘Other Models’ (Text S1). das Neves *et al.* also show a link between the total transcription rate 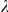 in a cell (measured through bromo-uridine incorporation across the whole nucleus) and 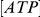, a sigmoidal curve, which we approximate (see ‘Parameterisation of 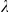
’ in Text S1) with
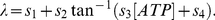
(2)


das Neves *et al.* record a change in the structure of this sigmoid curve in experiments where cellular chromatin is artifically decondensed. In these situations, the sigmoidal response of 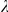 to 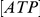 becomes a hyperbolic curve, with a sharp, continuous increase of 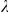 with 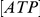 at low 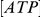. This change may reflect the necessity of remodelling chromatin – a process that requires ATP – for the transcription process. Chromatin remodelling has been noted by several studies [15], [16], [22] to play an important role in mRNA synthesis noise and hence downstream noise in gene expression. Rather than attempting to model this influence explicitly, we use the experimentally-determined form for 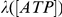 to capture the overall dependence of transcription rate (including chromatin effects) on 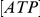.

To summarise, in our model, transcription rate depends sigmoidally on ATP concentration – a relationship elucidated and quantified in recent experiments [31]. ATP concentration in turn depends linearly on the mitochondrial mass and functionality level of a cell and also on the cell volume. Cells with many, highly functional mitochondria will have higher levels of ATP and hence higher transcription rates than those with smaller, less functional mitochondrial populations.

#### Cellular dynamics

Our model for cell cycle dynamics consists of equations governing the time evolution of the key quantities volume, mitochondrial mass, and mitochondrial functionality. In the light of a recent study [53], and as cell cycle models often assume the exponential growth picture, we expect an exponential form for cell volume growth: 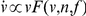. Here, 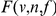 is a function expressing the dependence of volume growth rate on other parameters.

We suggest that ATP concentration (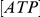) plays a key role in powering growth of the cell, so cells with higher ATP levels have higher growth rates associated with cell volume and mitochondrial mass. This link postulates that biosynthesis rates are generally, like transcription, a function of ATP concentration. We note that although ATP concentration has been suggested [31] as a possible mechanism linking mitochondria and transcription rate, and some evidence supports this link, it may be the case that a different factor provides the causal mechanism, and ATP concentration is correlated with this underlying factor. For example, ROS, which adversely affect many cellular processes (including provoking a decrease in transcription rates [31]), may be an alternative to ATP, or a combination of ATP and ROS levels may act to determine transcription rate.

Numerous historical studies, both in HeLa cells [54] and other tissue types [55]–[60] have found that the density 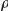 of mitochondrial mass (also called mitochondrial volume density) within cells of a given tissue type is consistent between generations and within populations. This consistency suggests that the time evolution of mitochondrial mass should be (a) coupled with the time evolution of volume and (b) of a form that allows damping of the inherent stochasticity at mitosis. In addition to these features, it is presumably reasonable to assume that mitochondrial growth is dependent on available 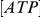 (due to the required protein synthesis). We suggest a model that captures these required dependencies and incorporates mean-reversion, given by the dynamic equations:
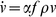
(3)

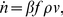
(4)where 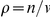.

We note that this simple model does not distinguish between volume growth rates at different times in the cell cycle, but yields a smooth exponential growth in cell size throughout the cell cycle. We work in this picture for simplicity and generality, but note that a more sophisticated model would include a more detailed description of cell growth as another potential source of variability between cells.

The model's dynamics result (see Methods) in a convergence in mitochondrial density with time to a value 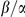.

#### Model parameterisation

**Figure 2:**
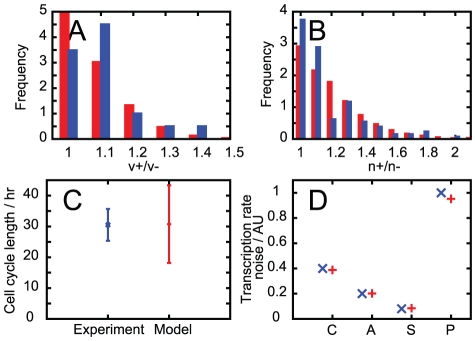
The set of data used to parameterise our model. Experimental data shown in blue, fitted simulated data shown in red. A. Ratio of larger cell volume to smaller cell volume between sisters at birth. B. Ratio of larger mitochondrial mass to smaller mitochondrial mass between sisters at birth. C. Mean and standard deviation of the cell cycle length in a population of cells. D. Noise levels in transcription rate in (C)ontrol, (A)ntioxidant-treated and (P)ro-oxidant-treated populations, and between (S)ister cells. Two other experimental values, not pictured, that were used to parameterise our model are a maximum cell volume of 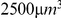 (for consistency with Ref. [53]) and a mean ATP concentration of 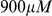 (from Ref. [70]).

Values for the parameters in our model were chosen (see Methods) to match a subset of experimental data, illustrated in Fig. 2.

### Our simple model is sufficient to approximate a large set of experimental data

Here we list a set of comparisons between predictions from our model and experimental studies. Unless stated otherwise, we will use experimental data from the study of das Neves *et al.* [31], using the protocol ‘NX’ to refer to data in Fig. X of that study.

#### Distributions of mitochondrial mass and cell volume

**Figure 3:**
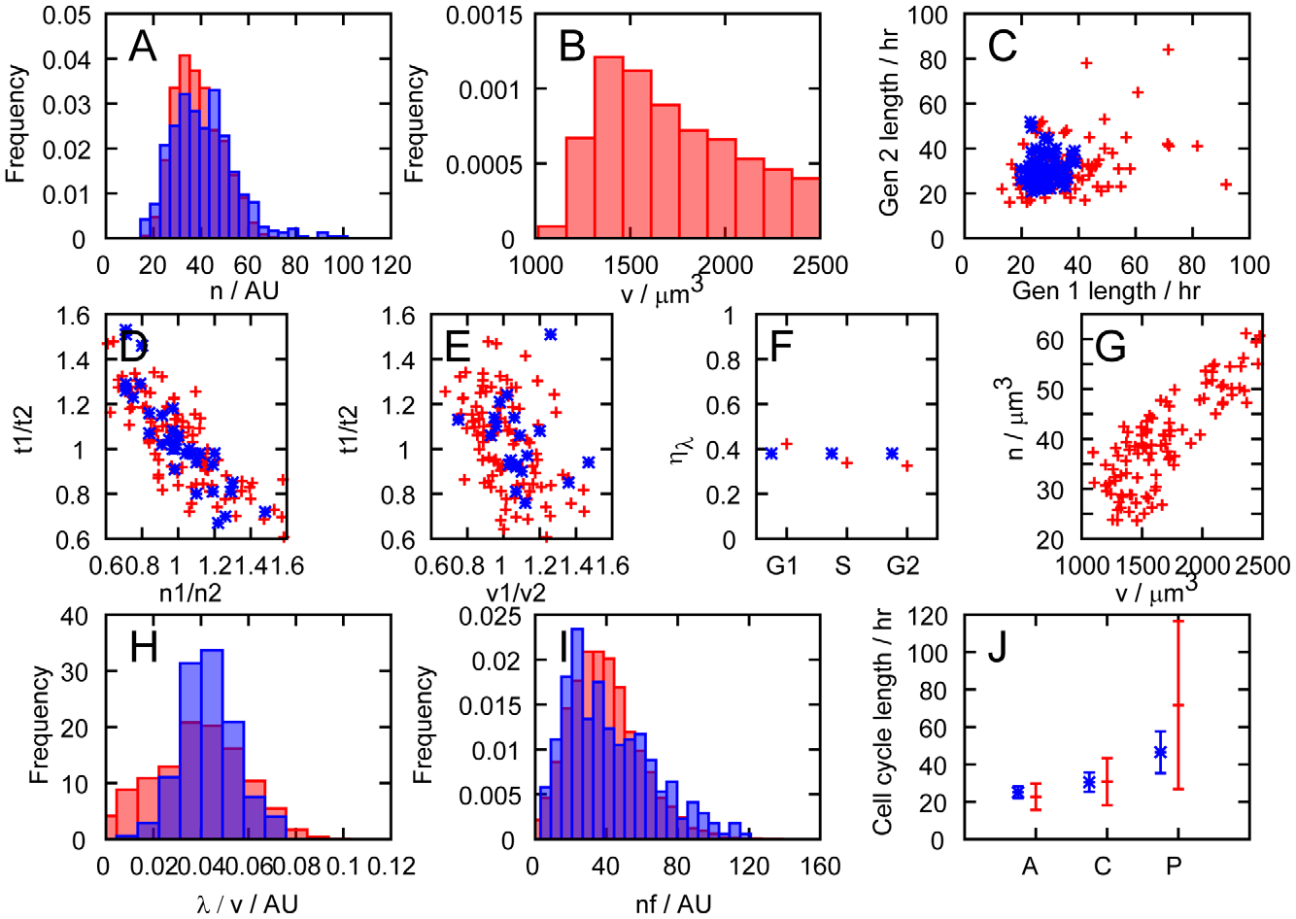
Our simple model is consistent with experimental probes of mitochondrial and cellular variability. Comparison between our model (red) and experimental data (blue), following discussion in the Main Text. **Experimental data from das Neves** ***et al.*** **** [**31**] **.** A. Distribution of mitochondrial mass 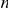 in an unsynchronised population of cells. B. Distribution of cell volume 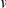 in an unsynchronised population of cells. C. Comparison of the lengths of cell cycles between generations: Gen 1 is the parent cell, Gen 2 the daughter. Cell cycle lengths are only weakly correlated. D. Relationship between the ratio of mitochondrial masses at birth against ratio of cell cycle lengths for sister pairs. E. Relationship between the ratio of cellular volumes at birth and the ratio of cell cycle lengths for sister pairs, showing a weaker correlation than D. F. Transcription rate noise 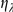 in subsets of the population in 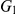, 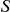, and 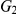 phases (see Main Text). G. Mitochondrial mass 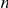 and cell volume 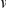 are strongly correlated in our model. Some experimental evidence is contradictory (see Main Text). H. Distribution of transcription rate per unit volume 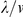. **New experimental data (see** **Methods** **).** I. Distribution of total mitochondrial functionality (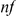 in our model, CMXRos readings from experiments). J. Mean and standard deviation of cell cycle lengths in (A)nti-oxidant-treated, (C)ontrol, and (P)ro-oxidant-treated populations. Experimental histograms, originally presented in arbitrary units, have been scaled to match the mean value of the simulated data.

Our model gives a peaked distribution skewed towards low 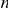 values for mitochondrial mass in the bulk population (Fig. 3A), which is similar in form to the experimental distribution (N4b). The distribution of cellular volumes in a bulk population (Fig. 3B) is found to display the quadratic decay expected from a theoretical treatment of cells growing exponentially [19].

#### Weak correlation between the lengths of successive cell cycles in a population

Fig. 3C shows the weak relationship between the cell cycle length of a parent and a daughter cell, which qualitatively matches experimental findings (from N4h).

#### Mitochondrial mass at birth is a better predictor of cell cycle length than cell volume at birth

Figs. 3D and 3E illustrate the correlations between cell cycle length and a cell's birth values of 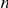 and 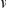 respectively. The correlation between birth mitochondrial mass and cell cycle length was strong (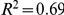, compared to the experimental value of 0.78) compared to the correlation between birth cell volume and cell cycle length (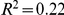, experimental value 0.22). The same correlation behaviour is observed in experiments (from N4e and N4f) which are shown for comparison.

#### Transcription rate noise with cell cycle stage

We modelled progression through the cell cycle stages by assigning stages according to the volume 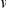 of a cell. We assign cells with 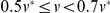 to 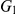, 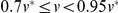 to 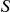, and 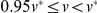 to 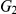 stages, to approximate the proportion of total cell cycle length that HeLa cells are observed to spend in each stage [61]. Transcription rate noise was found to stay relatively constant (around 0.4) when population subsets at different positions in the cell cycle were measured (see Fig. 3F), as observed in experiments (NS1).

#### Correlation between mitochondrial mass and cell volume

Our model predicts a strong correlation between cell volume 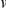 and mitochondrial mass 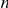 (Fig. 3G). This result contrasts with the weak correlation observed, using forward scatter in flow cytometry to measure volume, by das Neves *et al.* (N3a) (we confirmed these experimental results in this study – data not shown). However, many historic studies have found a much stronger connection between mitochondrial mass and cellular volume. The mitochondrial density 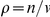, also referred to as mitochondrial volume density, has been found to exhibit low standard deviation (between 0.01 and 0.15 of the mean) in many different mammalian tissue types [54]–[60] and amounts of mtDNA have been found to display similarly low variability [55], [62]. These results contrast with the extremely high variability in mitochondrial volume density observed by das Neves *et al.* (the noise level estimated from the data is around 0.32), but we note that flow cytometry data (while useful for providing approximate orderings of cells by volume) may not be capable of providing the absolute volume measurements which are required to refute the low variability in 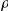 observed in many other studies.

#### Distribution of transcription rate per unit volume

Fig. 3H shows the distribution of transcription rate per unit nuclear volume (in our model, nuclear volume is taken as proportion to cell volume) in the bulk population. This result follows a similar peaked distribution to that found experimentally (N1a).

#### Others

**Figure 4:**
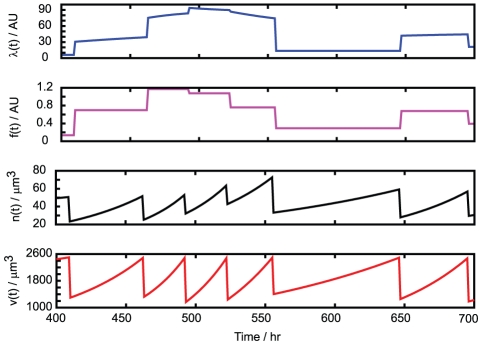
Illustration of the dynamics of our model. Example time series of 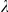 (transcription rate), 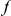 (mitochondrial functionality), 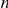 (mitochondrial mass) and 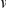 (cell volume), as a cell grows and divides repeatedly in our model.

We also note some qualitative features of our model: an increase in transcription rate with ATP levels is observed (trivially due to the functional form of 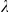), which is also observed experimentally (N3g). We also observe an increase in transcription rate per unit volume with total mitochondrial functionality (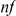 in our model), found experimentally (N3d). Fig. 4 shows illustrative time series of the dynamic variables involved in simulation of our model.

### New experimental results are also consistent with this model

In Fig. 3, we also present new experimental results pertaining to our model. These new experiments were designed to characterise two additional features of cells in a population: a measure of the total level of mitochondrial function within cells and the modulation of cell cycle lengths by changing the oxidative state of the cell. The total level of mitochondrial function is experimentally measured using the intensity of signal from CMXRos, a dye that stains mitochondria and accumulates according to membrane-potential, integrated over a whole cell (see Methods). This signal reports on the integrated membrane potential across the entire cell, combining measures of mitochondrial mass and functionality. The population distribution of this quantity is of interest in exploring the link between mitochondrial mass and functionality between cells.

The modulation of cell cycle length with cellular oxidative state was investigated by observing the distribution of cell cycle lengths in a control population of cells and in populations of cells after anti-oxidant (dithiothretiol) or pro-oxidant (diamide) treatments (see Methods). Our model incorporates oxidative status by modulating the mean level of mitochondrial functionality, so mitochondria function more readily in an environment with low oxidative stress than one with high oxidative stress. As mitochondrial functionality is tied in our model, through growth rate, to cell cycle length, we would expect cell cycle lengths to decrease upon anti-oxidant treatment and increase upon pro-oxidant treatment.

#### Distribution of mitochondrial functionality

Fig. 3I shows the distribution of total mitochondrial functionality in a population of cells. In our model, this distribution is just the distribution of the quantity 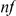, and in experiments, we measure the total membrane potential within a cell (see Methods). The predicted and experimentally observed distributions share a skewed form with similar variances.

#### Cell cycle lengths in different oxidative conditions

In Fig. 3J we show the mean and standard deviation of cell cycle lengths in a control population, and upon treatment with anti- and pro-oxidants (see Methods). In our simulations, these treatments are modelled by changing the value of 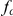, affecting the mean functionality of mitochondria (see Table 1). It is observed that treatment with anti-oxidants reduces cell cycle lengths, and treatment with pro-oxidants increases cell cycle lengths. In our model, this behaviour emerges from the dependence of the rate of volume growth on 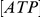, and the increased 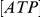 levels resulting from mitochondria with higher functionality.

**Table 1:** Parameters and values employed in our model.

Parameter	Description	Value	Motivation
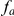	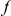 memory term	0.5	Fit parameter – chosen to give a mean functionality of 1
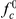	Sets mean functionality (control)	0.5	Fit parameter – chosen to give a mean functionality of 1
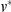	Volume for mitosis (scale)	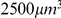	Fixed for consistency with maximum volume in Ref. [53]
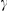	Proportionality between 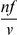 and 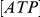	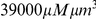	Fixed for consistency with mean ATP levels in Ref. [70]
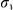	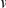 standard deviation at mitosis	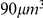	Fixed by volume segregation data in Ref. [31]
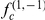	Set mean functionality (with anti-oxidant and pro-oxidant respectively)	(0.69, 0.09)	Fixed by transcription rate noise levels in Ref. [31]
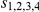	Fitting parameters for relationship between 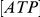 and 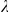	51.2, 44.7, 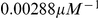 , 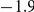	Fixed by functional form of 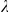 in Ref. [31]
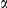	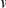 growth rate	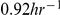	Chosen through optimisation – constrained by mean cell cycle length in Ref. [31].
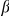	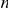 growth rate	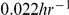	Fixed ratio with 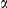 through mitochondrial segregation data in Ref. [31]
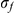	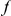 standard deviation at mitosis	0.34	Chosen through optimisation – constrained through transcription rate noise and cell cycle length variability in Ref. [31]

For further information see ‘Parameterisation of 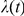
’ and ‘Fitting Other Parameters’ in Text S1.

#### Mitochondrial mass and membrane potential

We also observed a linear correlation between total mitochondrial mass (measured with MitoGreen) and total mitochondrial membrane potential (measured with CMXRos) in experiments performed with both dyes (see Methods and ‘Mitochondrial Membrane Potential’ in Text S1). This linear correlation emerges from our model due to our representation of total mitochondrial functionality as the product of a functional measure 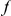 with mitochondrial mass 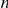. The observed correlation provides qualitative support for this representation.

### Summary of comparisons between experimental results and model predictions

It can be seen that several key experimental results require the inclusion of terms relating to mitochondrial variability for an explanation. In a situation without considerable mitochondrial influence on cellular variability, it may be expected that variability in cell cycle position among a population of unsynchronised cells may be a dominant source of noise. Physical distributions subject to such cell cycle noise would be expected to show a variance corresponding to an approximately twofold range, as this is the maximum difference in size between two unsynchronised cells. However, several results display data that varies over a considerably wider range than a factor of two, indicating that a factor other than cell cycle variability may be responsible. Most straightforwardly, Figs. 3A and 3I demonstrate pronounced cell-to-cell variability in the mass and functionality of mitochondrial populations. The distribution of transcription rate in Fig. 3I similarly shows a wide range of values.

Figs. 3D and 3E demonstrate the observed fact that mitochondrial inheritance at birth is a better predictor of cell cycle length than volume inheritance: an effect that relies on the presence of mitochondrial variability and mitochondrial influence on cellular growth. The variability in cell cycle length observed by modulating the oxidative state of the cell in Fig. 3J suggests that a source of variability that is sensitive to oxidative effects strongly affects cell cycle lengths. We believe that these results support the hypothesis that mitochondrial variability provides a significant contribution to the variability in distributions of the cellular properties we consider.

The correspondence between experimental data and the simulated behaviour of our model suggests that, although we have chosen simple functional forms in our model, the resulting behaviour is biologically relevant. However, we note here that our model was constructed from a phenomenological philosophy, with the intention of using experimental results to construct a plausible coarse-grained explanation for the influence of mitochondrial variability on extrinsic noise in general and transcription rate in particular: we were aware of all data from Ref. [31] when we were choosing the structure of our model though we only used a subset of available data to parameterise it. Our goal was to introduce a simplified but consistent mathematical summary of the data and to use this to motivate further experiments. To this end, we suggest a set of experiments in ‘Potential Experiments for Refinement’ (Text S1) that would support or contribute to further development of this model. We also note that many potential refinements could be made to our model and suggest several other functional forms in ‘Other Models’ (Text S1).

### Noise in transcription rate depends on noise in mitochondrial segregation and functionality

We are now in a position to explore the dependence of the level of noise in transcription rate on the stochasticity in mitochondrial mass and function, and subsequent stochasticity in 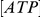. To investigate the contribution of mitochondrial variability to transcription rate noise, we performed simulations of our model while varying 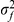, the variance associated with the inheritance of mitochondrial functionality, and 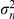, the variance associated with inheritance of mitochondrial mass. 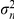 here gives the variance of the distribution by which mitochondrial mass is partitioned, and varying it under the assumption of binomial partitioning corresponds to changing the mitochondrial makeup of the cell: lower 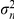 corresponds to more mitochondrial elements, each with smaller volume, while higher 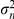 corresponds to fewer, larger mitochondrial elements, which are partitioned binomially at mitosis (see Methods).

**Figure 5:**
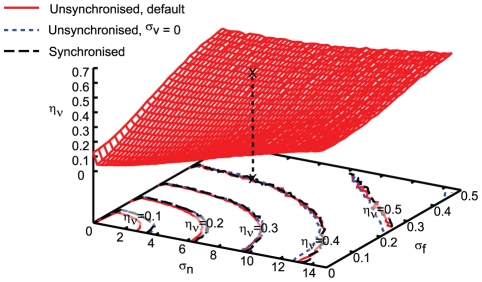
Variability in mitochondrial mass and functionality can both contribute to noise in transcription rate. Effects of changing variability in mitochondrial mass inheritance (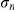) and functionality (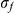) on overall transcription rate noise 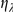. This contour plot shows the value of 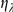 for a given combination of 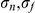. More stochasticity associated with inheritance of mitochondrial properties leads to higher transcription rate noise, and stochasticity in both mass and functional inheritance plays an important role in transcription rate noise. Contour lines on the bottom surface mark different values of 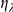. The ‘X’ mark denotes the default parameterisation of our model. Other contour lines show that this relationship remains essentially identical when variability due to cell cycle stage and volume inheritance is removed, suggesting that 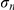 and 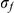 are the key sources of transcription rate noise.

In Fig. 5, the functional dependence of 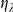 on mitochondrial variability (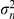 and 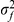) is shown from simulations. These results show that, for our model, the transcription rate noise is made up of significant contributions from both mitochondrial segregation and functionality. We also performed simulations where 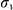, the variability arising from uneven volume partitioning, was set to zero, and where cells were sampled at the same position in their cell cycle, removing different ages as a source of variability. As Fig. 5 shows, the removal of these sources of variability has little impact on the overall transcription rate noise level. These results lead us to suggest that mitochondrial sources of variability provide a strong contribution to cell-to-cell variability in transcription rate. This argument is supported by an approximate analytic treatment of the sources of error in transcription rate within our model (see ‘Estimating Noise Contributions’ in Text S1).

### Mitochondrial variability can dominate noise in mRNA and protein expression

Having constructed and parameterised a model for mitochondrial variability and its effect on transcription in the cell, we now investigate the connection between these factors and downstream quantities: mRNA expression levels, and then (through further extension) protein expression levels. Noise in protein expression levels directly affects many cellular properties, as this noise causes cell-to-cell differences in the functional machinery available to perform cellular processes. Here we will investigate the influence of the mitochondrial variability suggested by the parameterisation of our model from experimental data on existing models for mRNA and protein expression. We connect our findings with the substantial existing body of literature on this topic in the Discussion section.

The production of mRNA and protein within a cell is often modelled using a master equation approach, addressing the probability of observing a given number of molecules at a given time. This analytical framework lends itself to the inclusion of our results for time-varying transcription rate (see Methods). Numerically, several studies have proposed techniques for incorporating time-varying rates in chemical kinetic systems [46], [47]: we use Shahrezaei *et al.*'s modification [45] to the Gillespie simulation method [63] to simulate our model system. This protocol allows us to investigate the relative importance of intrinsic contributions (resulting in differences in expression levels between identical genes within a single cell) and extrinsic contributions (resulting in differences in expression between identical genes in different cells in a population).

**Figure 6:**
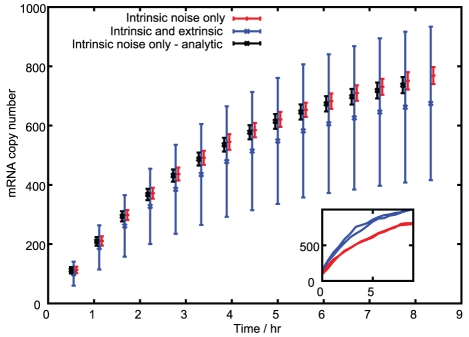
Mitochondrial variability contributes strongly to noise in mRNA levels. Analytic and modified Gillespie simulation results for time evolution of mRNA levels with and without mitochondrial and volume variability. Bars show the mean and standard deviation of the corresponding distribution at a given time. Red (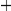) give simulated results without inherited variability. Black (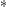) give analytic results without inherited variability. Blue (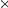) give simulated results with mitochondrial and volume variability, displaying much greater variance in mRNA expression. Bars are slightly offset in the x-direction for clarity. The inset shows two example time series for both simulated cases.

Fig. 6 shows the increase in mRNA expression (from a level of zero at the start of the simulation) from our analytic approach incorporating changing transcription rate, and in simulations run using (see Methods) a parameter set from Raj *et al.* [16], in two scenarios: one involving only intrinsic noise effects (no noise due to mitochondrial variability) and one involving extrinsic noise in transcription rate due to mitochondrial mass, functionality, and cell volume variability, of the magnitudes found through parameterising our model with experimental data. It can be seen that mitochondrial variability leads to a large increase in the total noise in mRNA expression levels: without extrinsic factors, the noise in mRNA expression at a given time (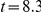 hours) was 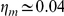, whereas 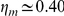 with extrinsic factors. We note that the means for the intrinsic and extrinsic noise cases differ: this result is due to the nonlinear dependence of transcription rate on ATP concentration, so that 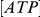 distributions with the same mean but different variances may yield transcription rate distributions with different means.

We can also perform simulations on the more complicated system involving protein production (see ‘mRNA & Protein Levels’ in Text S1). With values from Raj *et al.* [16] for protein degradation and translation rate (see Methods), this approach allows us to simulate dual reporter experiments, where the expression of two distinct but identically regulated protein-encoding genes is measured. Each protein was translated from a different mRNA strand, so these simulations tracked four quantities: the expression levels of the two mRNAs and the two proteins. Simulations were performed on synchronised and asynchronous cells, and with 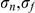 set to their model values and set to zero. In these simulations, mRNA molecules and proteins were also distributed binomially between daughter cells at mitosis (see Methods).

**Figure 7:**
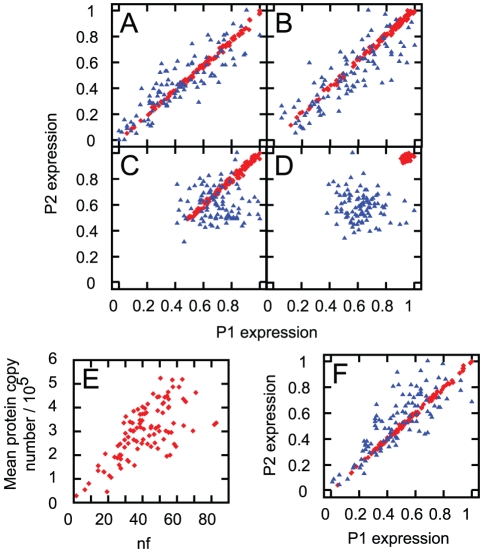
Effects of mitochondrial variability dominate protein expression variability in our model. Dual reporter simulation with different sources of noise in our protein expression simulations. All plots except (E) are normalised so that the highest protein expression level in the cell population is 1. Red (diamonds) show results from Raj *et al.*'s default parameterisation [16] used to model transcription, translation and degradation (see Methods). Blue (triangles) show results from this parameter set with degradation rates increased 100-fold. Protein levels are shown from population of (A) unsynchronised cells with mitochondrial and volume variability, (B) synchronised cells with mitochondrial and volume variability, (C) unsynchronised cells with no mitochondrial or volume variability, and (D) synchronised cells with no mitochondrial or volume variability. (E) Mean protein expression levels in the default parameterisation of Raj *et al.* with the product of mitochondrial mass and function 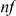, in the system corresponding to (A). (F) The equivalent plot of (A) with translation rates independent of 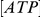.

Dual reporter simulations performed with the parameterisation chosen from Raj *et al.* [16] yield very low values for the magnitude of intrinsic noise. This low intrinsic noise was found to be due to the high copy number of proteins resulting from the parameterisation. To explore noise in systems with lower expression levels, we lowered the copy number of proteins by increasing the rates of mRNA and protein degradation (see Methods). Fig. 7 shows the resulting expression levels in two proteins with and without various sources of extrinsic noise, at the two different degradation rate protocols. These results show that, in our model, mitochondrial variability dominates the noise in protein expression levels. The spread of protein levels with mitochondrial and volume variability is much greater than the two-fold range achieved through cell cycle variability alone. Fig. 7 also illustrates that cells with higher mitochondrial mass and functionality generally have higher protein expression levels, though inheritance noise makes this correlation weaker.

In our model, we find that energy variability arising through mitochondrial stochasticity is the dominant source of variability in transcription rate, mRNA and protein expression levels. However, we note that the causal factors of stochasticity in mRNA and protein levels within the cell are significantly more complicated than the simple transcriptional model presented above. The rates of many of the processes involved in more extended models are functions of many factors which our model does not include. The inclusion of these complicating terms rapidly makes an analytic description of the model impossible. However, we note that stochastic simulation techniques may be used to explore the behaviour of complex model given estimates for the functional dependence of process rates on extrinsic variables [45].

We also note that several studies have observed a decrease in intrinsic noise at higher levels of protein expression [15], [27]. We do observe such a decrease, though in the default parameterisation the magnitude of this effect is very small owing to the consistently low intrinsic noise levels.

### Mitochondrial noise, by modulating transcription rate, can affect stem cell differentiation

As an illustrative application of our model, demonstrating its physiological relevance, we consider how, through the extrinsic effects of [ATP] on protein levels, a link between mitochondrial content and stem cell differentiation behaviour may arise. Differentiation dynamics in stem cells have often been modelled as the result of expression asymmetries in lineage regulation genes that interact in a regulatory network [64]–[67], but the initial sources of this expression variability have not been clearly elucidated and are a topic of active debate. Here we show that transcription rate variability resulting from mitochondrial variability can affect the dynamics of expression of such control genes. Experimentally, a link between stem cell differentiation and mitochondria was suggested by a recent study in mouse embryonic stem cells [38], showing that pluripotent cells with low mitochondrial membrane potential had higher *in vitro* differentiation propensity, whereas those with higher membrane potential remained undifferentiated and formed large teratomas.

We explore two recent models for the cell fate decision between erythroid and myeloid cell fates directed by the cross-antagonistic master lineage regulators GATA1 and PU.1. One model, by Huang *et al.* [68], consists of a symmetric coupled ODE system for the expression levels of these two genes, including cross-repression and self-activation term (see Methods). Another model, by Chickarmane *et al.* [69], contains a similar but asymmetric ODE model, expanded to include interactions with a postulated third species which is promoted by GATA1 and represses PU.1. The Chickarmane *et al.* model also includes external signalling terms which may act to promote GATA1 and PU.1, and repress the third species. In these models, cell states are defined by the relative levels of expression of these genes, such that undifferentiated cells have comparable levels of each transcription factor, while the two differentiated cell types correspond to a state with high levels of one factor and low levels of the other. The interactions between the genes are parameterised by variables such as self-activation and cross-repression rates (see Methods). The phase space for both these models comprises three attractor basins, corresponding to the progenitor cell type and two differentiated cell types.

**Figure 8:**
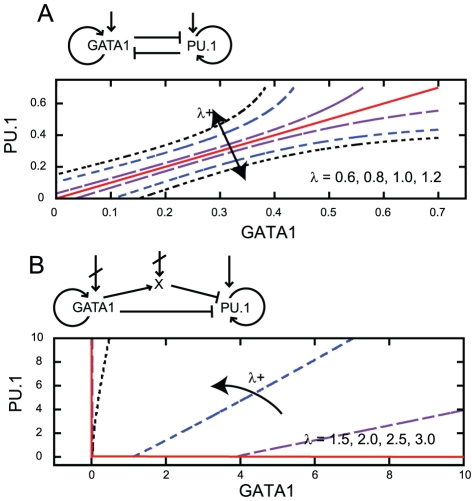
Transcription rate affects the stability of model stem cell systems. In both diagrams, curves delineate the boundary of the attractor basin corresponding to the undifferentiated cell state. Red (solid) to black (dotted) lines show the basin structure as transcription rate 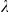 increases through the given values. (A) The structure of the undifferentiated attractor basin in the Huang model given different transcription parameters, showing the widening of the stable undifferentiated region at high transcription rate. (B) The structure of the undifferentiated attractor basin in the Chickarmane model, showing a decrease in undifferentiated basin size as transcription rate increases. The activation-repression structure of both models is illustrated – in (B), external terms representing the activation of GATA1 and X exist but are set to zero in our analysis to allow PU.1 to be expressed under some conditions.

Within the Huang model, at low protein expression levels, smaller perturbations are required to shift attractor basins than at high expression levels – a feature consistent across a large range of parameterisations. Varying the parameterisation of the model (modelling differentiation-inducing signalling) changes the structure of these basins, so that the central undifferentiated basin becomes more or less stable to subsequent perturbation. We vary the default parameterisation of the model in an attempt to assess the effect of changes in transcription and translation rates (see Methods). We find that when the parameters related to the rate of production of proteins are low, the central, undifferentiated state is less stable than when they are high (see Fig. 8A), with a smaller volume of phase space leading to the undifferentiated basin.

Within the Chickarmane model, a different effect is observed. As before, we investigated the volume of phase space corresponding to the basin representing the undifferentiated state. We used a nonzero value for the external signalling term promoting PU.1 and explored the system at different transcription rates (see Methods). We found that increasing the transcription rate led to a decrease in the range of values of the external interaction which supported a stable undifferentiated state (see Fig. 8B). This decrease in the stability of the undifferentiated state arose from a smaller volume of phase space leading to the undifferentiated basin as transcription rate increased, with more phase space occupied by the GATA1 basin. This result contrasts with the increased stability of the Huang model at high transcription rate, due to the importance of the third species (the expression of which is dependent on transcription rate): at high transcription rate, the increased strength of the combined effect of self-activating GATA1 and production of the third species shifts the basin structure strongly towards GATA1.

These results suggest that cell-to-cell variability in mitochondrial mass and function may, through induced variability in transcription rate, have a significant effect on the stability of bipotent cells. If differentiation dynamics are asymmetric and involve an intermediate species (as in the Chickarmane model), we find that high transcription rate destabilises the undifferentiated state. This destabilisation may be viewed as a result of the increased sensitivity of the system to perturbations: the asymmetric regulatory architecture means that a small increase in GATA1 will be quickly amplified at high transcription rate, as more GATA1 and X are quickly produced. If differentiation dynamics are symmetric and do not involve another species (as in the Huang model), high transcription rates increase the width of the basin corresponding to the undifferentiated state, acting to stabilise this state. This stabilisation is due to the increased robustness to perturbations afforded by the high production rate of both species at high transcription rate: without asymmetric interactions, the higher expression level of both genes makes the system less responsive to small perturbations. The results that emerge from this symmetric case gives results that are qualitatively comparable to an experimental study [38] in which more cells with higher total mitochondrial membrane potential remained undifferentiated, suggesting that high mitochondrial performance stabilises the undifferentiated state.

Another, higher-order effect may conceivably play a role in both situations: several studies have found that, at high protein abundance levels (which may result from high transcription rates), intrinsic noise levels in protein expression decrease. While the parameterisation of our dual reporter studies is such that these effects are small, the fact that less noise is expected at higher protein expression levels suggests a third mechanism by which high mitochondrial content may stabilise pluripotent cells. The contrasting results highlight the potential of experimental investigation of the effects of global transcription rate on the stability of multipotent states to inform of additional qualitative behaviors that models of lineage decision should be expected to exhibit.

## Discussion

We have introduced a crude mathematical model for the effects of stochasticity in mitochondrial segregation and functionality on transcription rate in cells. Our model, while simple enough to allow some analytic treatment, reproduces a good number of experimentally observed features concerning the interplay of mitochondrial properties and transcription rate. We analyse our model and find that mitochondria provide extrinsic noise contributions to transcription both through their uneven segregation at mitosis and through variability in their functionality.

We note that, in addition to requiring variability in the amount of mitochondrial mass, an adequate fit to our data required us to consider variability in the function of mitochondria. This connects with the wealth of recent experimental and theoretical interest regarding the causes and control of heterogeneity of mitochondrial function [34], [35], [42], [44] and strengthens the case for the broad physiological relevance of functional variability.

We incorporate our results for mitochondrial-sourced extrinsic noise into existing models for mRNA and protein production, and show that mitochondrial noise can lead to significant variability between cells in a population. We also suggest that transcriptional variability resulting from mitochondrial noise may affect stem cell differentation, and illustrate this result with an analysis of two recent regulatory network-based models for stem cell differentiation. We find that the quantitative effect of transcription rate variability on stem cell differentiation depends on the architecture of the regulatory network under consideration.

Several recent studies have investigated the interplay between other possible sources of extrinsic noise in various organisms. Before concluding, we will discuss connections to this body of literature. The recent study by Huh and Paulsson [29] found that variability in protein levels due to uneven inheritance at mitosis might explain a body of experimental data that was previously assumed to result from noise in the protein production process. A mathematical study by Rausenberger and Kollmann [21] also investigated the effects of inheritance stochasticity on cellular noise. Our work bears significant parallels to these ideas, in that we postulate uneven inheritance of mitochondria to be a substantial contributing factor to noise in all cellular processes that require ATP, including the mechanisms of protein production. Our philosophy also mirrors part of the work of Huh and Paulsson in that our model considers a subset of cellular properties (in our case, mitochondrial partitioning and functionality, and cell volume) to provide all stochastic influences, with all other cellular properties evolving deterministically.

The possible role of ATP as the proxy through which mitochondrial variability affects other cellular processes ties in with an early prediction of Raser and O'Shea [14] who suggested that the dominance of extrinsic noise in expression variability across a wide range of proteins could result from fluctuations in a factor that affects expression for all genes. ATP, being required for the processes of transcription and translation, meets this criterion. Shahrezaei *et al.* [45] illustrate the fact that extrinsic noise can influence intrinsic noise, through the former's effects on the rate constants involved in the latter. This influence plays an important role in our model, where extrinsic variability of mitochondrial properties influences the synthesis rates of mRNA and protein through their dependence on 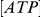. The ubiquity of ATP as an energy currency within the cell suggests that the rates of other intrinsic processes may be affected by the extrinsic variability we describe.

The link between the process of transcription and noise in protein expression levels that we explore in the last section of this paper is related to the findings of Blake *et al.* [22] who found that protein expression noise depends on transcription efficiency. In our model, the modulation of transcription rate by noisy 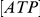 has downstream effects on protein noise levels.

Sigal *et al.* [26], in a study of expression levels over a range of proteins, find cell cycle stage to be a significant contributor to extrinsic noise in protein abundance. Volfson *et al.* [19] used a mathematical framework to similarly identify population dynamics, and upstream transcription factors, as key extrinsic contributors to cellular noise. Our model is compatible with these results, as cells at different cell cycle stages will have had different protein expression histories over their lifetimes. However, we anticipate that mitochondrial variability will also provide a significant contribution to protein expression noise, through modulation of upstream processes.

An in-depth study by Newman *et al.* in yeast cells [15] found a variety of protein-specific differences in expression noise according to transcription mode and protein function. Our model does not capture protein expression noise in this level of detail. The study of Newman *et al.* also characterised the contribution of intrinsic and extrinsic factors to total noise levels as a function of protein abundance. They found that while total expression noise did not scale with protein abundance, noise levels decreased with abundance when extrinsic factors were controlled for: suggesting that extrinsic factors were responsible for maintaining total noise levels as abundance increased. This suggestion that extrinsic noise increases in strength with protein abundance is captured in our protein level simulations.

A study by Bar-Even *et al.*, also in yeast cells [27], found intrinsic noise to be a substantial contributor to total noise, especially for proteins at intermediate abundance levels, with intrinsic contributions becoming less significant as expression levels increase (a similar result to Newman *et al.*). In this and several of the other studies above [15], [22], fluctuations in mRNA number were postulated to be the most important source of noise in protein expression levels. Our model suggests that, through the link between mitochondrial properties and transcription rate, mitochondrial variability strongly influences this important noise source and thus may be an important fundamental source of stochasticity in cellular biology.

Raj *et al.* [16] studied noise in mRNA expression in detail in mammalian cells (one of few studies to do so), and identified intrinsic effects as the dominant factors. Their study found that genes located in close proximity to each other displayed synchronised expression, while the expression of genes that were physically separate was unsynchronised, suggesting that local rather than global effects determine the expression levels of genes. While this study demonstrated that intrinsic effects significantly contribute to total noise in some cases, it was not explicitly shown that the magnitude of these effects outweighed extrinsic effects. Our results are compatible with this view that intrinsic noise plays an important role in gene expression, but we suggest that extrinsic noise due to energy variability may also be an important contributor to overall noise levels.

We do not attempt to capture these mRNA processes explicitly: rather, we take transcription rate to be a function of 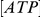 as found in experiments [31]. However, we note the result that the measured functional form of this relationship changes in experiments in which chromatin was decondensed. This result suggests that the functional form of transcription rate with 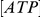 allows us to capture some effects of the ATP-dependent chromatin remodelling process.

### Conclusions

We find, through a phenomenological model constructed to reproduce recent data on mitochondrial and ATP variability, that stochastic inheritance of mitochondria at mitosis and variability in mitochondrial function may be important sources of noise in transcription. By extension, these factors may contribute significantly to noise in protein expression further downstream. We have proposed experimental tests to refine our model and demonstrate its application in existing models for mRNA and protein production and stem cell differentation, and discussed how these findings integrate into the current understanding of extrinsic noise in cellular biology. In particular, what our paper suggests is the need for multimodal single cell experiments through time (and through division) investigating coarse-grained measures of energy status, cellular volume, mitochondrial mass, and global rates of transcription and translation. Cellular variability is of central physiological importance but we suggest that to understand this we must elucidate the relationships between certain core variables, including the relationship between the machinery of expression and degradation and the energy status of the cell.

## Methods

### Parameterisation of our model from experimental data

We used a subset of available experimental data (shown in Fig. 2) to choose numerical values for our key parameters. Some parameter values were fixed, in the sense that maximising the agreement between predictions from our model and a single experimental study allowed an optimal value to be chosen straightforwardly (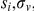 and the ratio 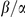 were parameterised by data from Ref. [31] (respectively the sigmoidal relationship between 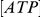 and transcription rate, the variance associated with volume partitioning, and the variance associated with mitochondrial partitioning), 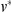 by the maximal cell volume observed in data from Ref. [53], and 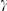 by comparing the mean properties of simulated cells to mean ATP levels reported in a population of HeLa cells in Ref. [70]). Other parameters influenced a range of predictions and values for these parameters were chosen by optimising the fit to experimental data across the set of results that they influenced (see ‘Fitting Other Parameters’ in Text S1).

Table 1 summarises the parameters and values employed in our model.

#### Model specifics

In the following we provide details of our model. The key equations specifying the key dynamics of cells in our model are given by Eqns. 1–4. The coupled ODEs in our model admit an analytic solution by simple integration:
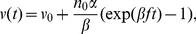
(5)

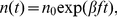
(6)The reader will note that a variety of models for mitochondrial and volume growth will yield similar forms. In addition, other functional forms for the level of ATP may be suggested. A selection of these alternative forms are explored in ‘Other Models’ in Text S1. Within this model, at mitosis,
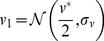
(7)

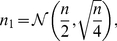
(8)where 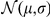 is a normal distribution with mean 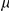 and variance 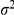, and 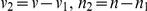 determine the daughter volumes 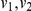 and mitochondrial masses 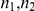. The variances of the mitochondrial distribution is chosen to represent a binomial distribution (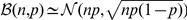, with 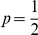, for high 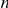). The variance of the volume distribution is chosen to match experimental data on volume partitioning.

Functionality evolves through changes at mitosis events, which follow an AR(1) process. A daughter cell's functionality 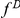 is determined from its parent's functionality 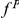:
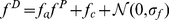
(9)Upon mitosis, both daughter cells inherit the same 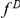 value, drawn from Eqn. 9. Once chosen, a cell's functionality remains constant throughout one cell cycle. We can model treatment with anti- and pro-oxidants by respectively increasing and decreasing 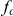, raising or lowering the mean functionality of mitochondria. With 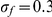, there is a very small but finite chance that cells will inherit zero (or lower) functionality. To avoid this unphysical case, we impose a cutoff of 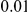 on mitochondrial functionality. Values under this cutoff are resampled from Eqn. 9. Another model, where 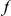 varies continuously within a cell cycle, is considered in ‘Other Models’ in Text S1.

### Virtual mitochondria

In our model, the standard deviation in mitochondrial mass at mitosis is a function of the number of virtual mitochondria in the cell, 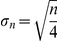, from binomial partitioning. We can vary the number of virtual mitochondria in the cell while keeping the total mitochondrial volume constant, by changing the volume assigned to an individual virtual mitochondrion. Let an individual virtual mitochondria have volume 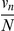, and let there be 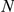 virtual mitochondria in a cell. The total mitochondrial volume is 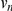, and the mean inherited mitochondrial volume is half of this. The standard deviation in virtual mitochondrial number from a binomial distribution of the virtual mitochondria is 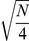, so the standard deviation of mitochondrial volume is 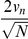. A standard deviation of 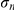 then corresponds to 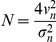 virtual mitochondria per cell.

### Cell dynamics simulations

To simulate a population of cells, we used a simple Euler method to solve the dynamic equations. A population of 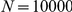 cells was simulated. When mitosis occurred, a random cell from the population was chosen for replacement by the new cell. To measure distributions, this procedure was continued until the distributions stabilised. To measure sister-sister and between-generation correlations, a list of relationships between cells was maintained, with sister pairs only sampled when both sisters underwent an entire cell cycle without replacement. We note that this removal of randomly-chosen cells may potentially introduce artefacts into the results, as the constant probability of cell removal means that the probability of a cell surviving to a certain age is decreasing. We checked our results with a different simulation protocol: running the system and allowing exponential growth up to 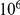 cells, with no removal. The resulting distributions were indistinguishable from the first protocol, showing that the removal rate is low enough so that the statistics are not affected.

### mRNA & protein expression simulations

For the simple system illustrating mRNA expression levels alone, cells were simulated using the modification of Shahrezaei *et al.*'s [45] to the Gillespie simulation method [63] in two scenarios: one involving only intrinsic noise (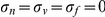) and one involving extrinsic noise due to mitochondrial mass, functionality, and cell volume variability. In both cases, the initial copy number of mRNA molecules was set to zero, to illustrate differences in the dynamics of the system: transcription started at the birth of the cell, and there were no effects from mRNA inheritance. In the intrinsic noise experiments, a population of cells were simulated from identical initial conditions, with their 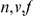 values set to the means of those variables obtained from simulations. The variability in mRNA expression levels in these cells was therefore solely due to intrinsic noise. For the extrinsic noise simulations, mRNA expression was simulated in the heterogeneous population of cells that resulted from dynamic simulation. An ensemble of 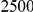 cells was analysed for both cases and the mRNA content at each timestep recorded.

To simulate the more complicated systems and investigate inheritance effects, we coupled the Shahrezaei *et al.* simulation protocol with the simple ODE solver so that simulation of a population of cells growing and producing mRNA and protein involved the following algorithm. 1) Use the ODE solver to calculate a cell's time of mitosis and the time series of volume and transcription rate throughout the cell lifetime. 2) Use Shahrezaei *et al.*'s method to compute the time behaviour of mRNA and protein levels given these time series for production rates. 3) Create daughter cells with noisy partitioning of volume, mitochondrial mass (binomial) and functionality (AR(1)), and mRNA and protein copy numbers (binomial).

After Raj *et al.* [16], we employ the following model values for birth (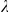) and death (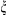) rates of mRNA (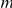) and protein (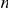): 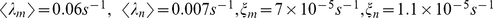. In the case of birth rates, we used these mean values to scale the 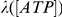 curves from das Neves *et al.* [31] so that the mean 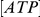 level observed in a population gave the mean 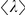 values from Raj *et al.* (see ‘mRNA & Protein Levels’ in Text S1). We also ran experiments with the degradation rates increased 100-fold: 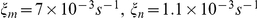, to explore the behaviour of the system at lower expression levels.

### Experiments

CMXRos labelling was done according to manufacturer (invitrogen) instructions, cells were incubated with the probe (75 nM) for 15 min. at 37 C and washed with warm PBS.

For the dual dye experiments, cells were loaded simultaneously with CMXRos and MitoTracker Green FM dye (Molecular Probes) for 20 min and after a brief PBS rinse fixed in PBS with 4% paraformaldehyde.

Cell cycle length experiments were done by growing cells in the presence of media alone or containing either the anti-oxidant Dithiothreitol (250 microM DTT) or the pro-oxidant Diamide (50 microM). Cell cycle length was measured as the time interval between two mitotic events in a single cell, analysed by live cell imaging using the Cell IQ platform and image analysis software (Chipman Tech.).

In the flow cytometry experiments, tripsinised Hela cells were washed and incubated with 20 nM MitoTracker Green FM dye (Molecular Probes) in medium at 37 C for 15 min, and then washed. Cells were analyzed using flow cytometry (Dako CyAn ADP).

### Master equations

Let us consider the master equation for the transcription process, describing the probability of observing the system with 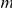 mRNAs at time 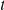:
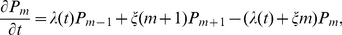
(10)where 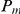 is the probability of observing 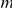 mRNAs at a given time 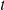, 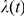 is transcription rate, and 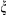 is an mRNA degradation rate.

Using a linear approximation for 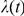 (see ‘Parameterisation of 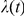
’ in Text S1), we can solve this by using a generating function approach (see ‘mRNA & Protein Levels’ in Text S1). The mean, variance, and probability distribution of mRNA copy number at arbitrary time are given by:
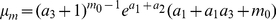
(11)

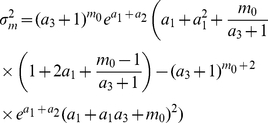
(12)

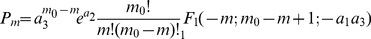
(13)where 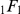 is the Kummer confluent hypergeometric function, 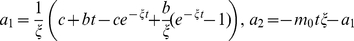 and 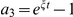.

### Stem cell model

The progenitor cell differentation model of Huang *et al.* [68] consists of the following equations for the evolution of protein expression levels 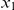 (GATA1) and 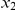 (PU.1):
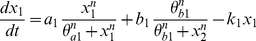
(14)

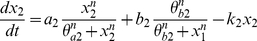
(15)In this parameter set, 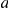 variables are self-activation rates, 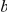 variables are cross-repression rates, 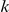 are decay rates, and 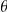 and 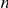 control the functional form of these processes. Huang *et al.* show that altering these parameters changes the structure of the corresponding attractor landscape, so that the central undifferentiated attractor basin changes in size, affecting the predisposition of the system to differentiate. This landscape change gives a ‘priming’ of the system such that the effect of subsequent asymmetries may vary. In this study, we vary the landscape by symmetrically varying 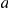 and 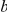, the parameters associated with activation and repression, from the default parameterisation 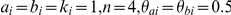 for 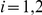. Specifically, we modulated 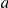 and 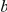 with a multiplicative factor 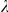: 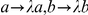. We draw the connection between higher values of 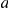 and 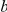 and higher transcription and translation rates, as the rate of production of chemical species is increased by an increase in these parameters.

The model of Chickarmane *et al.* [69] involves a relationshup between three dynamic variables:
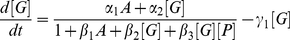
(16)

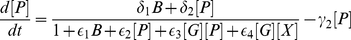
(17)

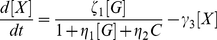
(18)where 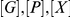 are the concentrations of GATA1, PU.1 and a postulated chemical species X respectively. 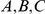 are external signalling factors: 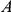 and 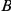 promote GATA1 and PU.1 respectively, and 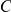 represses X. Other variables take default values 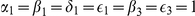, 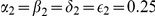, 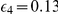, 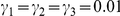, 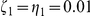, 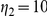. To vary transcription rates, we modulate 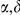 and 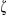 terms with a multiplicative factor which we take to be proportional to transcription rate 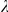. We used 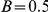 with 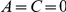, allowing an external signal that promotes PU.1, to promote stability of the undifferentiated state.

## Supporting Information

Text S1 Supplementary PDF document containing mathematical details of our model and alternative approaches to modelling the effects of mitochondrial variability.
(PDF)
