# Supplementary material for: Mitochondrial Variability as a Source of Extrinsic Cellular Noise
Source: PLoS Comput Biol. 2012 Mar 8;8(3):e1002416. doi: 10.1371/journal.pcbi.1002416 (PMC3297557; doi:10.1371/journal.pcbi.1002416)
Supplement: Text S1 — Supplementary PDF document containing mathematical details of our model and alternative approaches to modelling the effects of mitochondrial variability. (PDF) [file pcbi.1002416.s001.pdf]

Mitochondrial Variability as a Source of Extrinsic Cellular Noise – Supplementary Information

Parameterisation of $\lambda(t)$

Figs. 2g and 2i in das Neves *et al.* [1] give the response of transcription rate (λ) in arbitrary units to varying concentrations of ATP, without and with artificial decondensation of chromatin respectively. Measuring $[ATP]$ in μM and working with the same arbitrary units employed in that study, we model this response with the expression:

$$\lambda = s_1 + s_2 \tan^{-1}(s_3[ATP] + s_4), \tag{1}$$

with $s_1 \simeq 51.2, s_2 \simeq 44.7, s_3 \simeq 2.88 \times 10^{-3}\mu M^{-1}, s_4 \simeq -1.9$ (see Fig. 1).

das Neves *et al.* also produced a curve showing λ with $[ATP]$ in an experimental situation involving the decondensation of chromatin in the cell. This curve can be parameterised by the above equation with $s_1^d \simeq 40.0, s_2^d \simeq 54.7, s_3^d \simeq 1.6 \times 10^{-3}\mu M^{-1}, s_4^d \simeq -0.27$.

We choose this inverse tangent functional form to model the response of transcription rate to $[ATP]$ for mathematical simplicity in subsequent sections, and note that modelling with other functional forms (for example, Hill functions) is also possible.

In the parameterisation of our model, the time series of $[ATP]$ in cells is rather linear and slowly varying. This behaviour emerges both due to the dynamics of mitochondrial density (which tends towards $\frac{\beta}{\alpha}$ with time) and the fact that the exponential growths involved are cell-cycle limited to only span a factor of around two before mitosis. It will be useful to employ a linear approximation to $\lambda(t)$, gained from an expansion in t about t':

$$\begin{aligned} \lambda(t) &= s_1 + s_2 \tan^{-1}\left(s_4 + s_3 \frac{\gamma f n_0 e^{\beta f t}}{v_0 + \frac{n_0 \alpha}{\beta}(e^{\beta f t} - 1)} \right) && (2) \\ &\simeq c + bt, && (3) \end{aligned}$$

where the values of c and b are found after some algebra to be:

$$\begin{aligned} b &= \frac{s_2 f \beta t' d}{(1 + (s_4 + d)^2)} && (4) \\ c &= s_1 + s_2 \tan^{-1}(s_4 + d) - b, && (5) \end{aligned}$$

and

$$d = \frac{s_3 f n_0 \beta \gamma e^{\beta f t'}}{\alpha n_0 \left(e^{\beta f t'} - 1\right) + \beta v_0}. \tag{6}$$

Constants c and b then depend on cellular initial conditions and t' (which we take to be half the mean cell cycle length) but not on t. The sign of b is determined by the over- or under-population of mitochondria in the cell at mitosis: over-population will lead to high mitochondrial density and mean-reversion will act to decrease transcription rate with time (and *vice versa*).

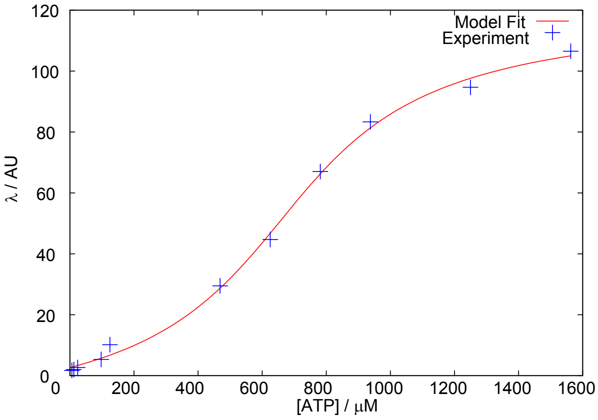

FIG. 1: λ with $[ATP]$ (experiment and fit).

Observable	Experimental Value	Simulated Value	Error
η_λ Control	0.4	0.389	0.028
η_λ Anti-oxidant	0.2	0.204	0.019
η_λ Pro-oxidant	1	0.943	0.057
η_λ Sister Cells	0.08	0.093	0.166
SD n_+/n_-	0.23	0.217	0.055
Cell cycle length	30.5	30.8	0.009
Mean $[ATP]$	900	925	0.028

TABLE I: Experimental observables and simulated fits obtained through optimisation of fitting parameters. Errors are the absolute difference between simulated and experimental values divided by experimental value. The score for a parameter set is the sum of errors for each observable.

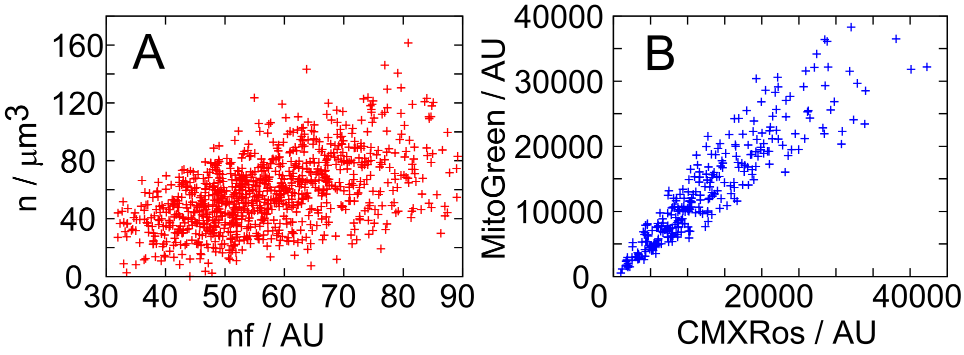

FIG. 2: (A) Simulated relationship between a measure of total membrane potential in a cell (the product of mitochondrial mass n and functionality f) and the mitochondrial mass in the cell. (B) Membrane potential (measured with CMXRos) against mitochondrial content (measured with MitoGreen).

Fitting Other Parameters

The parameters in our model concerning volume cutoff and partitioning were chosen straightforwardly: v^* for consistency with Ref. [2] and σ_v to best fit experimental data from Ref. [1]. We then needed to find values for the remaining parameters influencing properties of our model cells, namely α and β, the growth rates of cell volume and mitochondrial mass, γ, the constant of proportionality between $\frac{nf}{v}$ and $[ATP]$, and parameters determining the statistics of mitochondrial functionality f_a, f_c^0, σ_f. To fix values for these parameters, we first chose values for two parameters (f_a and f_c^0) to give a mean functionality of 1 in the control population, for simplicity. An optimisation procedure was then used to select values for σ_f, α, β and γ. The procedure we used involved choosing arbitrary initial conditions for each parameter (though the order of magnitude of these initial values was determined by a crude preliminary investigation) and iterating, choosing a new value for one of the free parameters at each step, and retaining this value if the overall performance of the parameter set improved. The new values were chosen either (with probability 0.1) uniformly over the order of magnitude associated with that parameter or (with probability 0.9) uniformly from the interval of 10% deviations from the old value. To judge performance, a score for each parameter set was calculated based on the absolute deviation between experimental and simulated observables, averaged over the seven quantities shown in Table I. Once these values were chosen for cells under 'normal' oxidative conditions (the default), $f_c^{-1,1}$ were set to match the experimentally observed transcription noise levels under the corresponding oxidative conditions.

Mitochondrial Membrane Potential

Fig. 2 shows a comparison of the relationship between total membrane potential in a cell and mitochondrial mass in the cell, from new experiments (see Methods in Main Text) and simulation of our model. Both our model and experimental data shows a linear correlation between total membrane potential and mitochondrial mass. This result emerges straightforwardly from our model due to the representation of total membrane potential (the product of n and f) and the qualitative agreement with experiment suggests that this modelling approach is suitable.

Estimating Noise Contributions

Here we find an expression for transcription rate in terms of the initial conditions and age of a cell, then estimate the variances associated with each of these contributory factors in order to assess the strength of each contribution to overall transcription

rate noise.

As cells evolve deterministically within a cell cycle, the concentration of ATP in a model cell is a function of the cell's initial conditions and its age:

$$[ATP] = \frac{\gamma f n}{v} \quad (7)$$

$$= \frac{\gamma f n_0 e^{\beta f t}}{v_0 + \frac{n_0 \alpha}{\beta}(e^{\beta f t} - 1)}, \quad (8)$$

where the second line follows from simple integration of our dynamic equations. If we assume that the initial conditions of an individual cell are uncorrelated – which is the case for single divisions within our model, but population and memory effects may possibly lead to correlations – we can use standard rules for combining means and variances to calculate the population mean and variance of the ATP distribution from the total differential:

$$\mu_{ATP} = \frac{\gamma \mu_f \mu_{n_0} e^{\beta \mu_f \mu_t}}{\mu_{v_0} + \frac{\mu_{n_0}\alpha}{\beta}(e^{\beta \mu_f \mu_t} - 1)}. \quad (9)$$

$$\sigma^2_{ATP} = \left|\frac{\partial ATP}{\partial n_0}\right|^2 \sigma^2_{n_0} + \left|\frac{\partial ATP}{\partial f}\right|^2 \sigma^2_f + \left|\frac{\partial ATP}{\partial v_0}\right| \sigma^2_{v_0} + \left|\frac{\partial ATP}{\partial t}\right|^2 \sigma^2_t \quad (10)$$

$$= \frac{e^{2ft\beta}\beta^2\gamma^2}{\left(\left(e^{ft\beta} - 1\right) n_0\alpha + v_0\beta\right)^4} \left(n_0^2 \left(n_0\alpha\left(e^{ft\beta} - 1 - ft\beta\right) + v_0\beta(1 + ft\beta)\right)^2 \sigma_f^2 \right.$$
$$\left. + f^2 v_0^2 \beta^2 \sigma^2_{n_0} + f^4 n_0^2 \beta^2 (n_0\alpha - v_0\beta)^2 \sigma_t^2 + f^2 n_0^2 \beta^2 \sigma^2_{v_0} \right) \quad (11)$$

$$= W^2_{n_0}\sigma^2_{n_0} + W^2_{v_0}\sigma^2_{v_0} + W^2_t\sigma^2_t + W^2_f\sigma^2_f. \quad (12)$$

As transcription rate λ depends solely on $[ATP]$ in our model, we can then calculate the mean and variance of the distribution of λ:

$$\mu_\lambda = s_1 + s_2 \tan^{-1}(s_3\mu_{ATP} + s_4) \quad (13)$$

$$\sigma^2_\lambda = \left|\frac{\partial \lambda}{\partial ATP}\right|^2 \sigma^2_{ATP} \quad (14)$$

$$= \left(\frac{s_2 s_3 \sigma_{ATP}}{1 + (\mu_{ATP}s_3 + s_4)^2}\right)^2. \quad (15)$$

From this, we can explore the contributions of mitochondrial segregation ($\sigma^2_{n_0}$) and functional diversity (σ^2_f) on transcription rate noise $\eta_\lambda = \frac{\sigma_\lambda}{\mu_\lambda}$. The overall expression can be written:

$$\eta_\lambda = \frac{\sigma_\lambda}{\mu_\lambda} \quad (16)$$

$$= \frac{s_2 s_3 \sigma_{ATP}}{(s_1 + s_2\tan^{-1}(s_3\mu_{ATP} + s_4))(1 + (\mu_{ATP}s_3 + s_4)^2)} \quad (17)$$

$$= \frac{s_2 s_3 \sqrt{W^2_{n_0}\sigma^2_{n_0} + W^2_{v_0}\sigma^2_{v_0} + W^2_t\sigma^2_t + W^2_f\sigma^2_f}}{(s_1 + s_2\tan^{-1}(s_3\mu_{ATP} + s_4))(1 + (\mu_{ATP}s_3 + s_4))^2} \quad (18)$$

$$= w\sqrt{w_f\eta_f^2 + w_{n_0}\eta^2_{n_0} + w_t\eta_t^2 + w^2_{v_0}\eta_{v_0}},, \quad (19)$$

where the last line condenses the expression into the quadrature sum of noise levels in cellular parameters with weighting factors $w_i = W_i^2\mu_i^2$.

Initial volume distribution. The mean and variance of initial volumes is simple to obtain, as division always occurs at a fixed volume and proceeds according to a fixed distribution. We straightforwardly have $\mu_{v_0} = \frac{v^*}{2}, \sigma_{v_0} = \sigma_v$.

Initial mitochondrial mass distribution. We estimate the population variance of initial mitochondrial mass as the variance associated with the daughter of a cell with population average properties just before mitosis. Such a cell has $v = v^*, n = \frac{\beta}{\alpha}v^*$. The mean and variance associated with initial mitochondrial mass in the daughter of such a cell is $\mu_{n_0} = \frac{\beta}{\alpha}\frac{v^*}{2}, \sigma_{n_0} = \sqrt{\frac{\beta v*}{4\alpha}}$. These values give a simple estimate comparing to numerical results on the population statistics (see Fig. 3A).

Mitochondrial functionality distribution. Similarly, we estimate the population distribution of mitochondrial functionalities by considering the daughter of a population-average cell. The steady-state distribution of the AR(1) process determining f is known to be normal with mean $\frac{f_c}{1-f_a}$ and variance $\frac{\sigma_f^2}{1-f_a^2}$. It is not straightforwardly obvious that the distribution of f in

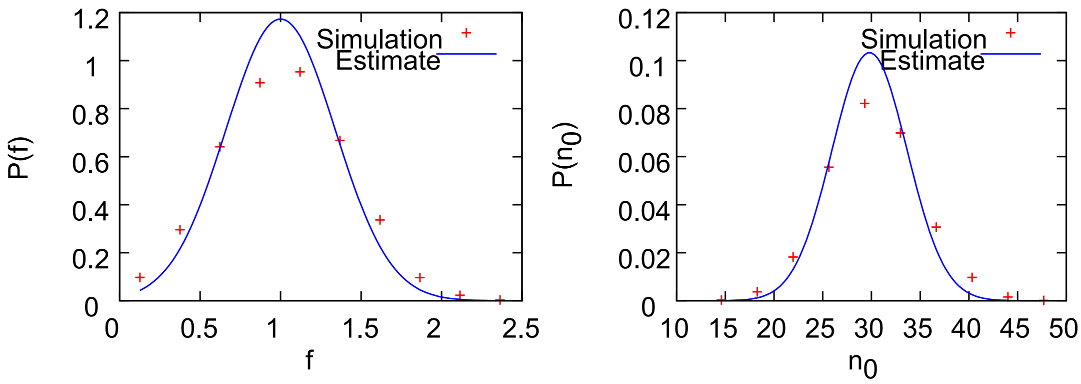

FIG. 3: A. Probability distribution of n_0 under default model parameters, compared to the estimated distribution from considering a population-average cell. B. Probability distribution of f under default model parameters, compared to the distribution of the underlying AR(1) process.

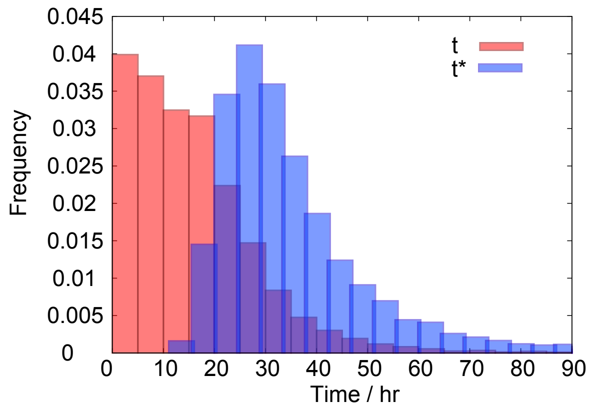

FIG. 4: Probability distributions of cellular age t and cell cycle length t^* under default model parameters.

an unsynchronised population of cells, where the lifespan of a cell is a function of f, should also follow this form, but numerical results confirm that the f distribution does closely match it (see Fig. 3B).

Distribution of cellular ages. This distribution is hard to estimate or approach analytically. We obtained the required values numerically from simulations, as shown in Fig. 4. The mean and variance of this distribution was found to vary with σ_{n_0} and σ_f, the variance in mitochondrial mass and mitochondrial function. Over the ranges $0 < \sigma_{n_0} < 20$ and $0 < \sigma_f < 0.4$, μ_t varied between 10 and 25 hours and σ_t varied between 9 and 50 hours. However, these terms provide a negligible contribution to the transcription rate variability (see later), so we approximate the mean and variance of $P(t)$ to be constant, with $\mu_t \simeq 16$ and $\sigma_t \simeq 14$, the values corresponding to the default model parameterisation.

We find, with our default parameter set, $w_f = 8.5 \times 10^5, w_{n_0} = w_{v_0} = 4.2 \times 10^5$, and that w_t is zero within working precision. Typical noise levels in these quantities were measured from simulations as $\eta_f = 0.34, \eta_{n_0} = 0.13, \eta_{v_0} = 0.07$. This approximate analytic approach thus supports the hypothesis that the dominant contributions to transcription rate variability are from mitochondrial variability terms.

Other Models

Continuous f

The behaviour of mitochondrial functionality f throughout and between cell cycles is the least well characterised property of our model. We use a simple picture in which f stays constant throughout a cell cycle and changes stochastically at mitosis, chosen for simplicity and due to the observed slowly varying behaviour of membrane potential in the cell cycle [1]. An alternative picture involves f being allowed to vary continuously and randomly throughout the cell cycle and no discrete jump occuring at mitosis, with daughter cells straightforwardly inheriting their parent's functionality. We found that this system was difficult to approach analytically, but performed numerical experiments to explore its behaviour. Instead of parameterising the behaviour of f with f_a and f_c, describing an autoregressive process, we now allow f to vary in a random walk with steps of size δf per unit time. δf then measures the degree of variability associated with f. To avoid unphysical situations, we place the constraint

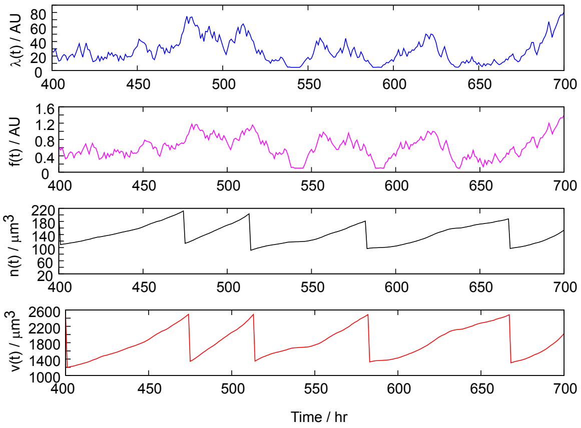

FIG. 5: Time series with varying f.

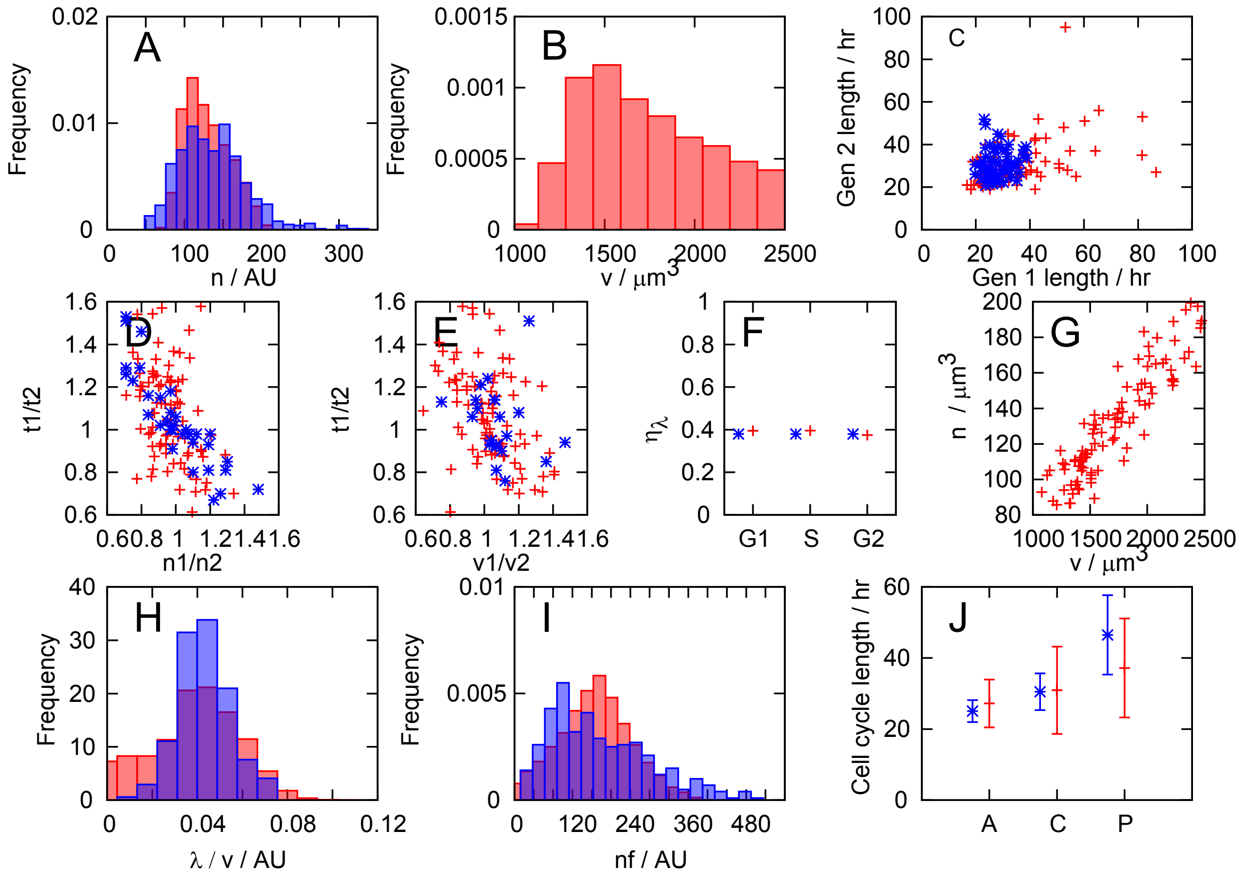

FIG. 6: **Behaviour of our model with continuously varying f.** Comparison to experimental data of the model with f continuously varying within cell cycles and inherited continuously at mitosis. Plots are the same as Fig. 4 in the Main Text.

$0.1 \leq f < 1.9$, forcing f to follow a constrained random walk.

Figs. 5 and 6 shows the behaviour of this model after parameterisation on the same data used to parameterise our default model. Most experimental results are captured to a similar extent than observed with our default model. These results suggest that the model's agreement with experimental data is robust with respect to the fine detail of the time evolution of f within cell cycles. This robustness suggests that it is the magnitude of extrinsic variability in mitochondrial functionality that gives rise to important physiological effects, rather than the specific detail of the time evolution and inheritance of functionality. However, as different dynamic regimes may adequately represent the behaviour of mitochondrial functionality, the need for further elucidation of mitochondrial functionality in model building is emphasised.

Label	Equations	Description
A	$\dot{v} = \alpha n$<br>$\dot{n} = \beta n$	Default model type
B	$\dot{v} = \alpha v$<br>$\dot{n} = \beta v$	Volume control on growth
C	$\dot{v} = \alpha n$<br>$\dot{n} = \alpha n \left(\gamma - \frac{n}{v}\right)$	Strong, explicit mean reversion of n towards a density γ.
D	$\dot{v} = \alpha v \left(1 - \frac{v}{v^*}\right)$<br>$\dot{n} = \alpha n \left(\gamma - \frac{n}{v}\right)$	Explicit mean reversion of n towards γ, and volume growth towards a target v^*
E	$\dot{v} = \alpha$<br>$\dot{n} = \beta n \left(\gamma - \frac{n}{v}\right)$	Linear volume growth
F	$\dot{v} = \alpha$<br>$\dot{n} = \beta \left(\gamma - \frac{n}{v}\right)$	Linear volume growth and weaker mean reversion on n

TABLE II: Different possible models for time evolution of the cell exhibiting mean reversion. Each of these expressions admits an analytic solution for $n(t)$ and $v(t)$. Other combinations (for example, weak mean reversion on n and exponential volume growth) are harder to solve and are often unstable for high or low initial n/v.

ATP and Alternatives

It is thought that ATP levels in the cell are subject to homeostasis. For this reason we do not include a sink term for ATP, assuming that an ATP deficit will be immediately compensated for. A model that would capture ATP usage is given by:

$$\dot{v} = \alpha' \frac{a}{v} \quad (20)$$

$$\dot{n} = \beta' \frac{a}{v} \quad (21)$$

$$\dot{a} = \gamma' n - (\alpha' + \beta') \frac{a}{v}, \quad (22)$$

where a is the cellular ATP level. Here, the second term in Eqn. 22 corresponds to the rate of use of ATP in increasing the cell volume and mitochondrial mass. Note that if $a = \gamma n$ our model equations are recovered. This model, with a suitable parameterisation, produces very similar results to our simpler model.

We also note that the following system explicitly incorporates ATP homeostasis, as ATP is produced up to a certain level by mitochondria and used up in the increase of cellular volume:

$$\dot{a} = \alpha'(a^* - a)n - \beta' a v, \quad (23)$$

where a^* controls the homeostatic level of ATP. This equation is solved by

$$a = a_0 e^{-t(\alpha' n - \beta' v)} + \frac{a^* \alpha' n}{\alpha' n + \beta' v}, \quad (24)$$

giving a hyperbolic form for ATP levels in terms of n and v. This representation could easily be extended to allow consideration of the ATP:ADP ratio – in which the rate of use of ATP gives the rate of production of ADP and vice versa.

It is also possible that ROS plays a role in modulating transcription rate and cellular growth rates. In this case, an additional term could be introduced into the model, proportional to the number of mitochondria, and possibly inversely proportional to mitochondrial functionality, and used to modulate the dynamic equations and transcription rate.

In our current model, we do not consider these complicating factors, due to our lack of experimental justification for them and also due to our desire to retain a simple, analytically tractable model. Future work, possibly motivated by the experiments we suggest in the main text, could take these more complicated factors into account.

Other Deterministic Dynamic Forms

Our model has been chosen phenomenologically to match experimental results concerning the distribution and behaviour of mitochondria. Other models are possible that display similar behaviour. In Table II we mention some potential alternative models. These alternatives both incorporate mean reversion on mitochondrial density and yield sensible key results. There is a difference in mean reversion rate between these models, illustrated by dynamic plots in Fig. 7.

Our model was chosen for its analytic tractability and its ability to reproduce experimental phenomena: these more complex models rapidly become analytically intractable, which hinders a deeper understanding of their behaviour.

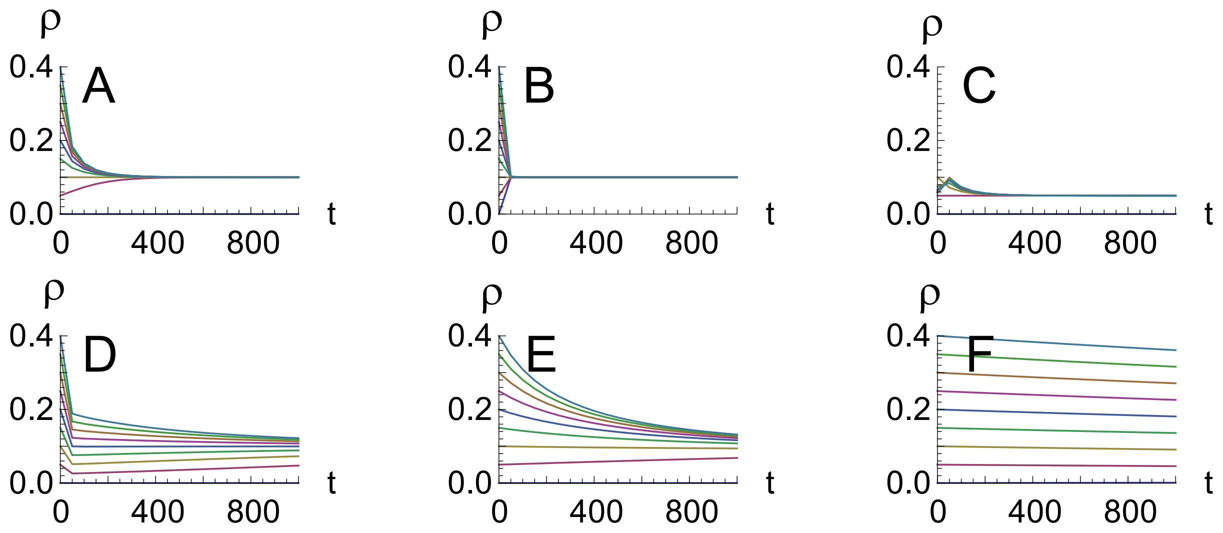

FIG. 7: The functional form of mitochondrial density ρ with time t (in arbitrary units) in other possible models for the time evolution of cellular properties. For these illustrations, the parameter set $\alpha = 0.1, \beta = 0.01, v_0 = 1000$ is used. The different lines correspond to trajectories of ρ resulting from varying initial n_0 between 0 and 400 (a much wider range than in the default model, as different choices of functional form may lead to different absolute values for n and v). The label of the plot gives the corresponding function in Table II.

We note in addition that the $[ATP]$ term that appears in our dynamic equations may be replaced by a transcription or translation rate term, explicitly including the sigmoidal (or hyperbolic) response of these cellular growth rates in the dynamic equations. In our bare model, we use the simpler $[ATP]$ term both to maintain analytic tractability and because the appropriate form for a response curve is difficult to estimate (for example, cellular growth may be a result of a combination of transcription and translation, and may include other additional terms). This approach essentially involves using a linear approximation for growth rate: linear approximations for transcription rate are employed elsewhere in this study and yield very similar results to the numerical behaviour of the corresponding nonlinear case.

It is possible to construct models for mitochondrial stochasticity that do not involve mean-reverting behaviour of mitochondrial density. As a simple example, if cellular dynamics are such that mitochondrial mass and cellular volume evolve independently:

$$\dot{v} = \alpha f v; \tag{25}$$

$$\dot{n} = \beta f n, \tag{26}$$

with mitosis involving a binomial segregation of volume and mitochondrial mass as before, the population variance in mitochondrial density will generally increase in an unbounded manner, as the probability of observing higher and higher mitochondrial densities increases with time. However, if cutoffs are placed on mitochondrial density, so that cells are removed from the population if their density does not obey

$$\rho_- < \frac{n}{v} < \rho_+, \tag{27}$$

the population distribution of mitochondrial density can achieve stationarity. This approach results in a much weaker correlation between mitochondrial mass n and cellular volume v. This model can be parameterised (an example parameter set is $\rho_- = 10, \rho_+ = 300, \alpha = \beta = 0.1, \gamma = 3.6$, other parameters the same as in the default model) to yield similar results to the default model for transcriptional noise.

This rather constructed model is considered due to the discrepancy between flow cytometry data in das Neves *et al.* [1], showing an absence of correlation between mitochondrial volume and cellular volume. Obtaining analytic results from this model is difficult, both in terms of approximation for the moments of probability distributions and the time evolution of mRNA.

We also note that, while our model results in cells with high mitochondrial density exhibiting high ATP levels, it could be the case that a different variable correlated with density is more fundamentally responsible for this relationship. For example, structural properties of the mitochondrial network within a cell – the largest connected component, or overall degree of connectivity – may be dependent on mitochondrial density and responsible for ATP levels.

Potential Experiments for Refinement

As mentioned in the Main Text, our model was constructed from a phenomenological philosophy, with the intention of using experimental results to construct a plausible coarse-grained explanation for the influence of mitochondrial variability on extrinsic

	Observable	Prediction
1	Mitochondrial functionality (measured via membrane potential) before and after mitosis.	Parent membrane potential is weakly retained but stochastically altered to give daughter membrane potential. Note that our current model treats this process crudely and as such many other dynamics may be possible (see 'Continuous f').
2	Protein expression levels as a function of $[ATP]$.	Expression levels are higher in cells with higher $[ATP]$.
3	Time series of mitochondrial mass and density through the cell cycle.	Exponential cell growth, with mitochondrial density tending towards an average value. The time series of these dynamics could be used to distinguish between different models (see 'Other Models').
4	Behaviour of $[ATP]$ with time.	Slowly varying over the cell cycle, mean-reverting towards an average value.
5	Determinant factors of $[ATP]$.	Mitochondrial mass and membrane potential, and cell volume. Levels of ROS may also be of importance.
6	Relative contribution of mitochondrial mass and functional variability to transcriptional variability (measured via bromouridine uptake).	Strong dependence of transcriptional noise on both mitochondrial inheritance and functionality variability.
7	Noise in protein expression levels.	Dependent on mitochondrial variability, and lower for cells with mitochondrial low mass and functionality.
8	ROS levels (measured via, for example, MitoSox).	Possibly correlated inversely with a measure of mitochondrial functionality, and possibly higher with low mitochondrial mass and functionality, as weaker/sparser mitochondria struggle to match energy demands.

TABLE III: Potential experiments that may clarify aspects of our model, roughly ranked in order of importance for supporting or suggesting area of refinement for our model.

noise in general and transcription rate in particular. Our goal was to introduce a simplified but consistent mathematical summary of the data and to use this to motivate further experiments. We suggest a set of experiments in Table II that would support or contribute to further development of this model.

mRNA & Protein Levels

The master equation for the probability distribution of mRNA levels can be written down as:

$$\frac{\partial P_m}{\partial t} = \lambda(t)P_{m-1} + \xi(m+1)P_{m+1} - (\lambda(t) + \xi m)P_m, \tag{28}$$

where $\lambda(t)$ is the time-dependent birth rate of mRNA molecules, ξ is the rate of removal of mRNA, and $P_m(t)$ is the probability of observing the system with m mRNA molecules at time t. We choose

$$\lambda(t) = (c + bt), \tag{29}$$

the approximation from 'Parameterisation of $\lambda(t)$', to model transcription rate with time.

Using the generating function $G(z) = \sum_m z^m P_m$ gives:

$$\frac{\partial G}{\partial t} = \lambda(t)(z-1)G - \xi(z-1)\frac{\partial G}{\partial z}. \tag{30}$$

We can solve Eqn. 30 with the method of characteristics. This process allows us to convert the PDE into a set of ODEs along a characteristic curve of the function. We wish to recast Eqn. 30 into the following form, where a characteristic curve is parameterised by the new variable s:

$$\frac{d}{ds}G(z(s), t(s)) = F(G, z(s), t(s)). \tag{31}$$

Using the chain rule, we can write:

$$\begin{aligned} \frac{d}{ds}G(z(s),t(s)) &= \frac{\partial G}{\partial z}\frac{dz}{ds} + \frac{\partial G}{\partial t}\frac{dt}{ds} && (32)\\ &= \frac{1}{z-1}\frac{\partial G}{\partial t} + \xi\frac{\partial G}{\partial z} = (c+bt)G, && (33) \end{aligned}$$

where the last line is just a rearrangement of the original PDE. By comparing coefficients, we obtain the characteristic equations for the system:

$$\begin{aligned} \frac{dz}{ds} &= \xi \rightarrow z = \xi s + z_0 && (34)\\ \frac{dt}{ds} &= \frac{1}{z-1} = \frac{1}{\xi s + z_0 - 1} \rightarrow t = \frac{1}{\xi}\ln(\xi s + z_0 - 1) + t_0, && (35)\\ \frac{dG}{ds} &= (c+bt)G = \left(c + b\left(\frac{1}{\xi}\ln(\xi s + z_0 - 1) + t_0\right)\right)G. && (36) \end{aligned}$$

These equations may be interpreted as describing the evolution of the arguments of G, and the subsequent evolution of the value of G, as we progress along a curve parameterised by s. As absolute values of s are not important, affecting only the parameterisation of progress along a characteristic curve, we set $z_0 = 0$ without loss of generality. We then have $s = \frac{z}{\xi}$ and $t_0 = t - \frac{1}{\xi}\ln(z-1)$. The final ODE can then be solved by separation of variables:

$$\begin{aligned} \int \frac{1}{G}dG &= \int \left(c + b\left(\frac{1}{r}\ln(\xi s + z_0 - 1) + c_2\right)\right) ds && (37)\\ &= s(c + bt_0) - \frac{sb}{\xi} + \frac{sb}{\xi}\ln(\xi s - 1) - \frac{b}{\xi^2}\ln(\xi s - 1) && (38)\\ &= \frac{z}{\xi}(c+bt) - \frac{bz}{\xi^2} - \frac{b}{\xi^2}\ln(z-1), && (39) \end{aligned}$$

We then have

$$G = C\exp\left(\frac{z}{\xi}(c+bt) - \frac{bz}{\xi^2}\right)(z-1)^{\frac{-b}{\xi^2}}, \quad (40)$$

where the arbitrary function $C = C(t - \frac{1}{\xi}\ln(z-1)) = C(t_0)$, as this quantity is independent of s.

If the initial copy number of mRNA molecules is m_0, we have the initial condition $G(z,0) = \sum_m z^m P_m(0) = \sum_m z^m \delta_{m\, m_0} = z^{m_0}$. Noting that at $t = 0$, $e^{-t_0\xi} = z - 1$, we find that if we employ the choice

$$C(t_0) = \exp\left((e^{-t_0\xi}+1)\left(\frac{b}{\xi^2} - \frac{c}{\xi}\right)\right)(e^{-t_0\xi}+1)^{m_0}\exp\left(\frac{-b}{\xi^2}t_0\xi\right), \quad (41)$$

we recover the required initial condition:

$$\begin{aligned} G(z,t_0) &= \exp\left(\frac{z}{\xi}(c+bt) - \frac{bz}{\xi^2}\right)(z-1)^{\frac{-b}{\xi^2}}\exp\left((e^{-t_0\xi}+1)\left(\frac{b}{\xi^2} - \frac{c}{\xi}\right)\right)e^{-t_0\xi}+1)^{m_0}\exp\left(\frac{-b}{\xi^2}t_0\xi\right) && (42)\\ G(z,t_0=0) &= \exp\left(\frac{zc}{\xi} - \frac{bz}{\xi^2}\right)(z-1)^{\frac{-b}{\xi^2}}\exp\left(z\left(\frac{b}{\xi^2} - \frac{c}{\xi}\right)\right)z^{m_0}(z-1)^{\frac{b}{\xi^2}} && (43)\\ &= z^{m_0}. && (44) \end{aligned}$$

The general solution is then given by Eqn. 42, which, using $t_0 = t - \frac{1}{\xi}\ln(z-1)$, can be written:

$$G(z,t) = e^{a_1 z + a_2}(z + a_3)^{m_0}, \quad (45)$$

with

$$\begin{aligned} a_1 &= \frac{1}{\xi}\left(c + bt - ce^{-\xi t} + \frac{b}{\xi}(e^{-\xi t} - 1)\right) && (46)\\ a_2 &= -m_0 t\xi - a_1 && (47)\\ a_3 &= e^{\xi t} - 1 && (48) \end{aligned}$$

We can recover the mean and variance of the distribution $P_m(t)$ by using $\mu_m = \frac{\partial G}{\partial z}\big|_{z=1}$ and $\sigma_m^2 = \frac{\partial^2 G}{\partial z^2}\Big|_{z=1} + \frac{\partial G}{\partial z}\big|_{z=1} - \left(\frac{\partial G}{\partial z}\big|_{z=1}\right)^2$:

$$\mu_m = (a_3+1)^{m_0-1} e^{a_1+a_2}(a_1 + a_1 a_3 + m_0) \tag{49}$$

$$\sigma_m^2 = (a_3+1)^{m_0} e^{a_1+a_2}\left(a_1 + a_1^2 + \frac{m_0}{a_3+1}\left(1 + 2a_1 + \frac{m_0-1}{a_3+1}\right) - (a_3+1)^{m_0+2} e^{a_1+a_2}(a_1 + a_1 a_3 + m_0)^2\right) \tag{50}$$

We can also recover the full probability distribution of observing m mRNAs using $P_m(t) = \frac{1}{m!}\frac{\partial^m G}{\partial z^m}\big|_{z=0}$. Using Leibniz's rule allows us to write:

$$P_m(t) = \frac{1}{m!}\frac{\partial^m G}{\partial z^m}\Big|_{z=0} \tag{51}$$

$$= \frac{1}{m!}\sum_{i=0}^{m}\binom{m}{i}\frac{\partial^i}{\partial z^i}e^{a_1 z + a_2}\frac{\partial^{m-i}}{\partial z^{m-i}}(z+a_3)^{m_0}\Big|_{z=0} \tag{52}$$

$$= \sum_{i=0}^{m}\frac{1}{i!(m-i)!}a_1^i e^{a_1 z + a_2}\frac{m_0!}{(m_0-m+i)!}(z+a_3)^{m_0-m+i}\Big|_{z=0} \tag{53}$$

$$= \sum_{i=0}^{m}\frac{m_0! a_1^i e^{a_2} a_3^{m_0-m+i}}{(m_0-m+i)! i! (m-i)!}. \tag{54}$$

This can be alternatively written as

$$P_m(t) = a_3^{m_0-m} e^{a_2}\frac{m_0!}{m!(m_0-m)!}\,{}_1F_1(-m; m_0-m+1; -a_1 a_3) \tag{55}$$

where ${}_1F_1$ is the Kummer confluent hypergeometric function.

The corresponding master equation including proteins is:

$$\frac{\partial P_{m\,n}}{\partial t} = m\lambda_n(t)P_{m\,n-1} + \xi_n(n+1)P_{m\,n+1} - \xi_n n P_{m\,n} - m\lambda_n(t)P_{m\,n} + \lambda_m(t)P_{m-1\,n} + \xi_m(m+1)P_{m+1\,n} - \xi_m m P_{m\,n} - \lambda_m(t)P_{m\,n}, \tag{56}$$

for which we have not obtained a general analytic solution. Instead, we simulate this system with a stochastic simulation algorithm. To simulate these systems, we use a parameter set employed by Raj *et al.* [3]: $\langle\lambda_m\rangle = 0.06\,s^{-1}$, $\langle\lambda_n\rangle = 0.007\,s^{-1}$, $\xi_m = 7\times10^{-5}\,s^{-1}$, $\xi_n = 1.1\times10^{-5}\,s^{-1}$. The death rates are simply inserted into the simulation, and the mean birth rates are used to tune the time-varying birth rate for the simulation. For example, the normalisation of $\lambda_m(t)$ was chosen so that the population average value of $[ATP]$ corresponded to $\langle\lambda_m\rangle$.

As the process of translation is believed to be $[ATP]$-dependent but not dependent on chromatin remodelling, the decondensed version of Eqn. 1 (using the hyperbolic s_i^d coefficients rather than the sigmoidal s_i coefficients) was used for λ_n. Again, the mean birth rate was used to normalise this function as above. Overall, we then have constant rates for mRNA death (faster) and protein death (slower) and time-varying birth rates for mRNA (including chromatin remodelling) and proteins (not including chromatin remodelling).

As mentioned in the Main Text, we found this parameterisation to yield large copy numbers of proteins, which led to very low values for intrinsic noise. We explored this effect by increasing the degradation rates from Raj *et al.*'s default values, to $\xi_m = 7\times10^{-3}\,s^{-1}$, $\xi_n = 1.1\times10^{-3}$. In addition, we investigated the case where variability in $[ATP]$ only affected transcription rate, with translation rate taking the constant value $\lambda_n = 0.007\,s^{-1}$. As shown in the Main Text, those simulations with higher degradation rates showed a more significant intrinsic noise contribution, and the trends in these dual reporter simulations changed little with a constant translation rate. This consistency means that though our crude model of the dependence of translation rate on $[ATP]$ could be challenged, even if translation is ATP dependent we still see pronounced effects on protein levels through ATP-dependent variability in transcription rate.

[1] R. P. das Neves, N. S. Jones, L. Andreu, R. Gupta, T. Enver, and F. J. Iborra. Connecting Variability in Global Transcription Rate to Mitochondrial Variability. *PLoS Biol.*, 8(12):451–464, 2010.

[2] A. Tzur, R. Kafri, V. S. LeBleu, G. Lahav, and M. W. Kirschner. Cell growth and size homeostasis in proliferating animal cells. *Science*, 325(5937):167–171, 2009.

[3] A. Raj, C. S. Peskin, D. Tranchina, D. Y. Vargas, and S. Tyagi. Stochastic mRNA synthesis in mammalian cells. *PLoS Biol.*, 4(10):e309, 2006.
